# Predicting five-year comorbid bipolar disorder after attention-deficit/hyperactivity disorder diagnosis: a population-based machine learning approach

**DOI:** 10.1186/s13034-025-01002-3

**Published:** 2025-12-01

**Authors:** Yen-Shan Yang, Chih-Wei Hsu, Liang-Jen Wang, Kuo-Chuan Hung, Yang-Chieh Brian Chen, Chih-Sung Liang, Mu-Hong Chen

**Affiliations:** 1 Department of Psychiatry, Kaohsiung Chang Gung Memorial Hospital, Chang Gung University College of Medicine, No. 123, Dapi Road, Niaosong District, Kaohsiung, 833 Taiwan; 2 Department of Child and Adolescent Psychiatry, Kaohsiung Chang Gung Memorial Hospital, Chang Gung University College of Medicine, Kaohsiung, Taiwan; 3 Institute for Translational Research in Biomedicine, Kaohsiung Chang Gung Memorial Hospital, Chang Gung University College of Medicine, 833401 Kaohsiung, Taiwan; 4 Department of Anesthesiology, Chi Mei Medical Center, Tainan, Taiwan; 5 Department of Psychiatry and Behavioral Sciences, The University of Texas Health Science Center at Houston, Houston, TX USA; 6 Department of Psychiatry Tri-Service General Hospital, National Defense Medical University, Beitou branch, Taipei, Taiwan; 7 Department of Psychiatry, National Defense Medical University, Taipei, Taiwan; 8 Department of Psychiatry, Taipei Veterans General Hospital, No. 201, Shih-Pai Road, Sec. 2, Taipei, 11217 Taiwan; 9 Department of Psychiatry, College of Medicine, National Yang Ming Chiao Tung University, Taipei, Taiwan

**Keywords:** ADHD, Artificial intelligence, BD, Feature, Interpretation, Prediction

## Abstract

**Background:**

Early detection and accurate prediction of bipolar disorders (BDs) comorbidity in individuals with attention-deficit/hyperactivity disorder (ADHD) are clinically critical. This study used machine-learning methods to identify features predictive of subsequent BD among patients initially diagnosed with ADHD.

**Methods:**

We analyzed claims from the Taiwan National Health Insurance Research Database (2000–2013) and included patients aged ≥ 12 years with at least two diagnoses of ADHD. Predictor features included demographics (sex, age at ADHD onset), healthcare utilization (psychiatric outpatient visit counts), comorbidities (International Classification of Diseases–coded diagnoses), psychiatric medications (Anatomical Therapeutic Chemical–coded prescriptions), and family psychiatric history. All features were extracted from prespecified windows around the ADHD diagnosis date (index date). The primary outcome was a subsequent BD diagnosis. We trained an extreme gradient boosting (XGBoost) classifier and tuned hyperparameters via grid search to maximize the area under the receiver operating characteristic curve (AUROC). Feature importance was interpreted with Shapley additive explanations (SHAP).

**Results:**

Among 15,093 eligible patients, 266 (2%) developed BD during follow-up. The model achieved a ROC-AUC of 0.90 and a precision–recall AUC of 0.59; accuracy was 98%, specificity 99%, sensitivity 50%, and positive predictive value 43%. Twelve leading predictors emerged. The strongest behavioral signal was sparse psychiatric visits before ADHD diagnosis followed by frequent visits afterward (SHAP = 0.27 and 0.66, respectively). Core demographic risks were older age at ADHD onset (SHAP = 0.26) and male sex (SHAP = 0.08). Medication pattern included pre-diagnosis short-acting benzodiazepines (SHAP = 0.07) and post-diagnosis exposure to anticonvulsant mood stabilizers (SHAP = 0.34), “-dones” (SHAP = 0.06) and “-pines” (SHAP = 0.05) antipsychotics, selective serotonin-reuptake inhibitors (SHAP = 0.06), and Z-drugs (SHAP = 0.05). Protective features were having offspring with schizophrenia-spectrum disorders (SHAP = 0.11) and fewer new-onset upper-respiratory infections after ADHD diagnosis (SHAP = 0.06).

**Conclusions:**

Leveraging nationwide real-world data, we built a machine-learning model to predict subsequent comorbid BD in patients with ADHD. The identified clinical and medication prescribing profiles can alert clinicians to patients at heightened risk, facilitating earlier monitoring and timely intervention.

**Supplementary Information:**

The online version contains supplementary material available at 10.1186/s13034-025-01002-3.

## Background

The early identification of bipolar disorder (BD) in individuals with attention-deficit/hyperactivity disorder (ADHD) is critical, particularly since ADHD usually presents in childhood and BD in adolescence or adulthood [1]. Longitudinal cohorts suggest that ADHD symptoms may represent a prodromal phase of BD, and approximately 5–12% of people with ADHD subsequently receive a BD diagnosis [2, 3]. When ADHD and BD co-occur, symptom burden intensifies, resulting in greater inattention, hyperactivity, and impulsivity for ADHD [4], and more unstable mood states during manic or hypomanic episodes for BD [5]. This is accompanied by markedly elevated risks of substance misuse, anxiety disorders, and other mental-health complications [6, 7]. However, the two disorders share overlapping features (impulsivity, pressured speech, irritability, distractibility, and increased motor activity), often complicating differential diagnosis [8, 9]. Against this backdrop, delineating risk factors for comorbid BD in routine care could facilitate earlier monitoring and intervention, potentially improving outcomes for this vulnerable population.

Pharmacological exposures, heightened mental-health service utilization, and immune-inflammatory comorbidities have all been implicated in accelerating the progression from ADHD to BD. In a cohort of 4869 patients with ADHD who initiated antipsychotics (e.g., risperidone, quetiapine, aripiprazole), the incidence of comorbid BD within 1 year was as high as 21% [10]. Recurrent psychiatric hospitalizations and emergency-department visits are likewise emerging as early warning signals of diagnostic conversion [11], although the independent predictive value has yet to be rigorously quantified. Immune-related conditions appear influential as well: adolescents with comorbid asthma showed a 31-fold elevated risk of BD risk, far surpassing the roughly ten-fold increase observed in ADHD alone [12]. Despite these robust epidemiological links, only a handful of investigations have translated such associations into clinically actionable prediction tools. Given the serious consequences of delayed or missed BD diagnoses, early detection and accurate risk stratification within ADHD populations remain essential for optimizing long-term outcomes.

Recent advances in machine-learning techniques have shown considerable promise in predicting clinical outcomes [13]. By applying feature-engineering methods, such models allow clinicians to identify ADHD patients who are at elevated risk of developing BD at an earlier stage. In this study, we combined nationwide population data with machine-learning algorithms to build predictive models for subsequent BD comorbidity in patients with ADHD. These models incorporated real-world features, including patient demographics, patterns of healthcare service use, comorbid medical and psychiatric conditions, and psychiatric medication records. Moreover, we systematically evaluated and ranked the importance of individual features to enhance model interpretability and to provide practical clinical insights for risk-stratified management.

## Methods

### Study design

This research received approval from the Chang Gung Memorial Hospital Institutional Review Board (No. 202300262B0). We conducted a retrospective cohort analysis using the Taiwan National Health Insurance Research Database (NHIRD), which has covered >99% of the Taiwanese population since 1996 [14]. The NHIRD provides de-identified enrollment files, demographic characteristics (sex, date of birth, family linkage), dates and diagnoses of all outpatient and inpatient encounters, and details of dispensed prescriptions [14]. Each beneficiary is assigned a unique encrypted identifier, allowing longitudinal follow-up while protecting personal privacy.

**Fig. 1:**
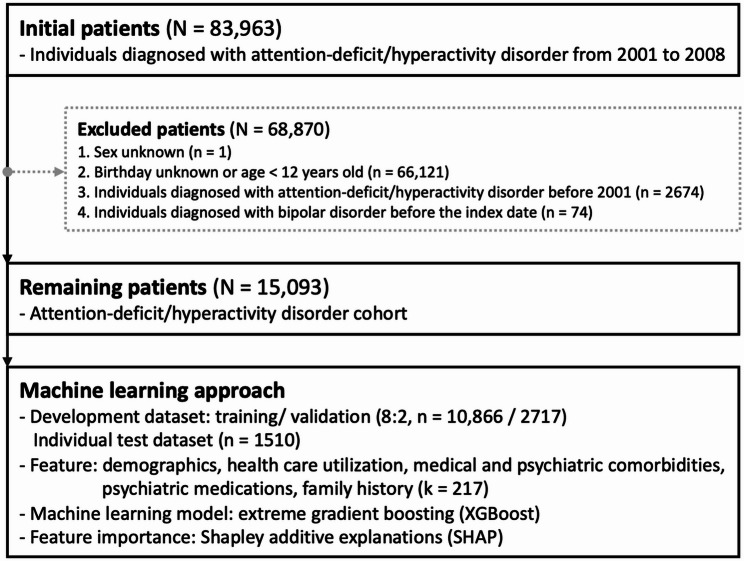
Flowchart of the selection process for this study

Figure 1 summarizes the cohort-selection process and the model development process. We retrieved NHIRD claims from 1 January 2000 to 31 December 2013; all diagnoses were coded according to the International Classification of Diseases, Ninth Revision, Clinical Modification (ICD-9-CM). The reliability of observational studies based on NHIRD data has been demonstrated in prior research [15, 16]. Cohort assembly proceeded in several steps. First, we identified individuals who received ≥ 2 psychiatrist-confirmed diagnoses of ADHD (ICD-9-CM 314) between 1 January 2001 and 31 December 2008. The date of the first qualifying ADHD diagnosis served as the index date, which is consistent with the operational definition widely used in Taiwanese studies [17, 18]. Second, we excluded patients whose sex or birth date was missing or whose age at the index date was < 12 years, because a five-year follow-up beginning before age 12 would fall short of the typical window for BD onset. Third, we removed patients with any ADHD diagnosis recorded before 2001 to ensure a full one-year look-back period for capturing baseline comorbidities and psychotropic prescriptions. Fourth, we excluded anyone with a BD diagnosis (ICD-9-CM 296.0, 296.1, 296.4–296.7, 296.80, 296.81, 296.89) before the index date. All remaining ADHD cases were followed up for exactly five years to ascertain incident BD. A uniform five-year horizon was possible because enrollment ended on 31 December 2008 and NHIRD data extended through 31 December 2013, guaranteeing identical observation windows for every participant. After applying these criteria, the final analytic cohort comprised 15,093 patients with ADHD.

## Study variables

The primary outcome was the first recorded diagnosis of BD occurring during the five-year follow-up from each patient’s ADHD index date. To build predictive models for this outcome, we extracted several features from the NHIRD [19, 20]. First, demographic characteristics—sex and age at ADHD diagnosis—were documented. Second, healthcare utilization was quantified as the mean number of psychiatric outpatient visits and inpatient admissions during the year preceding the ADHD index date and during the year immediately preceding either the BD diagnosis date or the end of follow-up (index date + five years); for individuals with ADHD and BD diagnoses separated by less than 12 months, visit and admission counts were prorated to a 12-month equivalent to preserve comparability. Third, comorbidities were captured as newly recorded medical or psychiatric conditions within the same one-year windows; once a patient was assigned a given diagnosis, subsequent entries for that condition were not considered new onset. Fourth, we evaluated psychiatric medications, which are grouped into antidepressants, antipsychotics, mood stabilizers (including anticonvulsants and lithium), and benzodiazepines (including Z-drugs). Unlike comorbidities, the use of psychiatric medications was recorded based on the patient’s condition at a specific time, and we did not limit this classification to newly initiated treatments. If a patient used any medication from a given category at any time within the specified windows—excluding time after the BD diagnosis date (for cases) and the end-of-follow-up date (for non-cases) to prevent information leakage—they were classified as a user of that category. Fifth, family psychiatric history was identified if any first- or second-degree relative (parents, offspring, siblings, grandparents, or grandchildren) had a psychiatric diagnosis, applying the same criteria used for patient comorbidities. A total of 217 features were found in this study, and details on the precise definitions of comorbidities and medications are presented in eTable 1.

## Machine learning model

After feature extraction, we randomly partitioned the cohort into a 90% development set and an independent 10% test set (final performance reporting). The development set was then split 80/20 into training subset (model fitting) and a validation subset (performance monitoring) [21, 22]. We used the development set to train an extreme gradient-boosting (XGBoost) classifier to predict subsequent comorbid BD in patients with ADHD [23]. XGBoost is an ensemble of gradient-boosted decision trees that sequentially fits trees to residuals via gradient descent and uses explicit regularization to limit overfitting [24, 25]. It flexibly models non-linear relationships and higher-order interactions, handles missing values natively, and scales well to high-dimensional feature spaces. In tabular prediction tasks [24, 25], XGBoost has frequently achieved state-of-the-art accuracy and often outperforms deep neural networks [26]. Hyperparameters were tuned within the training subset by grid search to maximize the area under the receiver operating characteristic curve (ROC-AUC) on the validation subset. We fixed baseline settings (number of trees: 100; learning rate: 0.1; balance between positive and negative classes: 59) and searched over maximum depth of each decision tree (3–10), minimum sum of instance weight needed in a child (1–10), subsample (0.5–1), subsample ratio of columns when constructing each tree (0.5–1), and gamma (0–0.5). The selected configuration was then locked, and performance—ROC-AUC, precision–recall AUC (PR-AUC), accuracy, specificity, sensitivity, and positive predictive value (PPV)—was evaluated on the independent test set. To assess the robustness of the model performance metrics, we conducted a sensitivity analysis using a bootstrap-like resampling approach. Specifically, we randomly divided the development set into a training set and a validation set, repeating this process 10 times to generate 10 independent models. Subsequently, we evaluated the performance of each model on the original independent test set. Finally, we calculated the mean values with 95% confidence intervals for all performance metrics across all models to quantify the variability and stability of our results.

We used Shapley Additive Explanations (SHAP) to explain each prediction [27]. The model has a baseline when no features are considered; adding a person’s features pushes the prediction up or down. SHAP assigns, for each person and feature, the average contribution under a fairness rule that averages over all possible feature orders. The “SHAP index” (global importance) is the average size of a feature’s per-person SHAP contributions across the independent test set, indicating typical influence regardless of sign. Positive values raise predicted risk; negative values lower it. As SHAP values are additive, the total prediction can be decomposed into the sum of the individual contributions of each feature. We conducted two exploratory sensitivity analyses. First, to assess the relative informational contribution of temporal windows, we performed temporal ablations: (1) removed all pre-index features (healthcare utilization, comorbidities, psychotropic medications), retaining post-index features only (Ablation-PostOnly); and (2) removed all post-index features, retaining pre-index features only (Ablation-PreOnly). Training and evaluation procedures were otherwise identical. Second, to examine age-dependent heterogeneity, we fit age-stratified models by age at ADHD diagnosis—Age-Stratum-12–25 and Age-Stratum-≥30—chosen to align with two major BD onset windows [28, 29]. All analyses were conducted in SAS software (version 9.4), and machine-learning procedures, including XGBoost training and SHAP computation, were implemented in Python (version 3.10, using the scikit-learn and XGBoost libraries).

## Results

Table 1 summarizes baseline characteristics for the development dataset (training subset, *n* = 10,866; validation subset, *n* = 2717) and the independent test set (*n* = 1510). In the largest analytic sample, the training subset, the mean age at ADHD diagnosis was 18.6 years, 30% of participants were female, and 1.7% had comorbid BD during the five-year follow-up period. During the 12 months preceding the index date, patients averaged 13.5 psychiatric outpatient visits, whereas in the final 12 months of the five-year observation window, the mean fell to 11.4 visits. No psychiatric hospitalizations were recorded for any patient during both observation periods. Regarding medical comorbidities observed prior to ADHD diagnosis, the most frequent new-onset conditions included upper respiratory infection (12.6%) and asthma (2.6%). Conversely, the most prevalent new-onset psychiatric disorders were depressive disorders (4.7%) and anxiety disorders (3.0%). After ADHD diagnosis (one year before the end of observation date), the most frequently observed new-onset conditions were also upper respiratory infection (18.2%) and asthma (3.8%). Correspondingly, the most common new-onset psychiatric disorders identified were depressive disorders (6.8%) and anxiety disorders (5.7%). Regarding psychotropic drug exposure, the most commonly prescribed subclass within each drug category can be summarized as follows. Before ADHD diagnosis, 2.4% of participants received anticonvulsant mood stabilizers (the leading mood-stabilizer subclass), 4.6% received first-generation antipsychotics (the leading antipsychotic subclass), 11.0% received selective serotonin-reuptake inhibitors (the leading antidepressant subclass), and 10.1% received short-acting benzodiazepines (the predominant benzodiazepine subclass). After ADHD diagnosis, the corresponding prevalences were 2.6% for anticonvulsants, 4.5% for first-generation antipsychotics, 5.1% for selective serotonin-reuptake inhibitors, and 9.4% for short-acting benzodiazepines, indicating that these subclasses remained the foremost agents within their respective categories throughout the study period. eTable 3 additionally presents these characteristics stratified by comorbid BD status.

**Table 1:** Characteristics of development and test datasets

Characteristics	Development		Test
	Training (*n* = 10,866)	Validation (*n* = 2717)	Test (*n* = 1510)
Basic information			
Age at first diagnosis of attention-deficit/hyperactivity disorder	18.6 ± 10.0	18.8 ± 10.0	18.2 ± 9.3
Sex, female	3276 (30.1)	825 (30.4)	457 (30.3)
Psychiatric outpatient visit			
Number of annual visits before the index date	13.5 ± 10.3	13.5 ± 10.6	13.3 ± 10.0
Number of annual visits before the end date	11.4 ± 10.9	11.7 ± 11.1	11.3 ± 10.9
Medical comorbidities (Pre–index date)			
Upper respiratory infection	1364 (12.6)	320 (11.8)	184 (12.2)
Asthma	285 (2.6)	74 (2.7)	45 (3.0)
Psychiatric comorbidities (Pre–index date)			
Depressive disorders	512 (4.7)	125 (4.6)	60 (4.0)
Anxiety disorders	322 (3.0)	86 (3.2)	37 (2.5)
Medical comorbidities (Post–index date)			
Upper respiratory infection	1979 (18.2)	498 (18.3)	270 (17.9)
Asthma	418 (3.8)	101 (3.7)	62 (4.1)
Psychiatric comorbidities (Post–index date)			
Depressive disorders	744 (6.8)	205 (7.5)	201 (6.7)
Anxiety disorders	622 (5.7)	147 (5.4)	76 (5.0)
Psychotropic drugs (Pre–index date)			
Mood stabilizers			
Anticonvulsants	259 (2.4)	51 (1.9)	30 (2.0)
Lithium	23 (0.2)	6 (0.2)	4 (0.3)
Antipsychotics			
First-generation	512 (4.7)	125 (4.6)	58 (3.8)
The benzamides	404 (3.7)	112 (4.1)	48 (3.2)
The -dones	174 (1.6)	35 (1.3)	29 (1.9)
The -pines	72 (0.7)	13 (0.5)	10 (0.7)
Antidepressants			
Selective serotonin reuptake inhibitors	1205 (11.1)	300 (11.0)	159 (10.5)
Serotonin–norepinephrine reuptake inhibitors	153 (1.4)	37 (1.4)	25 (1.7)
Anxiolytics, sedatives, or hypnotics			
Long-acting benzodiazepines	1061 (9.8)	276 (10.2)	122 (8.1)
Short-acting benzodiazepines	1098 (10.1)	284 (10.5)	148 (9.8)
Z-drugs	513 (4.7)	134 (4.9)	70 (4.6)
Psychotropic drugs (Post–index date)			
Mood stabilizers			
Anticonvulsants	279 (2.6)	72 (2.6)	34 (2.3)
Lithium	25 (0.2)	4 (0.2)	1 (0.1)
Antipsychotics			
First-generation	487 (4.5)	129 (4.7)	56 (3.7)
The benzamides	204 (1.9)	60 (2.2)	27 (1.8)
The -dones	117 (1.1)	34 (1.3)	21 (1.4)
The -pines	114 (1.0)	22 (0.8)	12 (0.8)
Antidepressants			
Selective serotonin reuptake inhibitors	556 (5.1)	758 (5.8)	93 (6.2)
Serotonin–norepinephrine reuptake inhibitors	99 (0.9)	34 (1.3)	8 (0.5)
Anxiolytics, sedatives, or hypnotics			
Long-acting benzodiazepines	823 (7.6)	219 (8.1)	112 (7.4)
Short-acting benzodiazepines	1020 (9.4)	148 (9.1)	230 (8.6)
Z-drugs	406 (3.7)	108 (4.0)	51 (3.4)
Psychiatric family history			
Attention-deficit/hyperactivity disorder			
Parents	214 (2.0)	55 (2.0)	26 (1.7)
Siblings	1194 (11.0)	278 (10.2)	183 (12.1)
Offspring	1014 (9.3)	250 (9.2)	124 (8.2)
Schizophrenia spectrum disorders			
Parents	151 (1.4)	39 (1.4)	34(2.3)
Siblings	97 (0.9)	23 (0.9)	24(1.6)
Offspring	27 (0.3)	8 (0.3)	2(0.1)
Bipolar disorders			
Parents	144 (1.3)	39 (1.4)	28(1.9)
Siblings	94 (0.9)	17 (0.6)	22(1.5)
Offspring	20 (0.2)	3 (0.1)	3(0.2)
Information of outcomes			
Bipolar disorder comorbidity during follow-up	185 (1.7)	51 (1.9)	30 (2.0)
Age at the diagnosis of bipolar disorder	24.5 ± 12.0	21.6 ± 7.1	23.5 ± 9.4

Data was expressed as N (percentage) or mean ± standard deviation

Final model hyperparameters were: number of trees = 100, learning rate = 0.1, balance between positive and negative classes = 59, maximum depth of each decision tree = 10, minimum sum of instance weight needed in a child = 10, subsample = 1, subsample ratio of columns when constructing each tree = 0.5, and gamma = 0.2. On the independent test set (*n* = 1510), the model achieved a ROC-AUC of 0.90 and a precision–recall AUC (PR-AUC) of 0.59; accuracy was 98%, specificity 99%, sensitivity 50%, and PPV 43%. eTable 4 lists the detailed performance metrics and bootstrap resampling sensitivity analysis for all datasets.

**Fig. 2:**
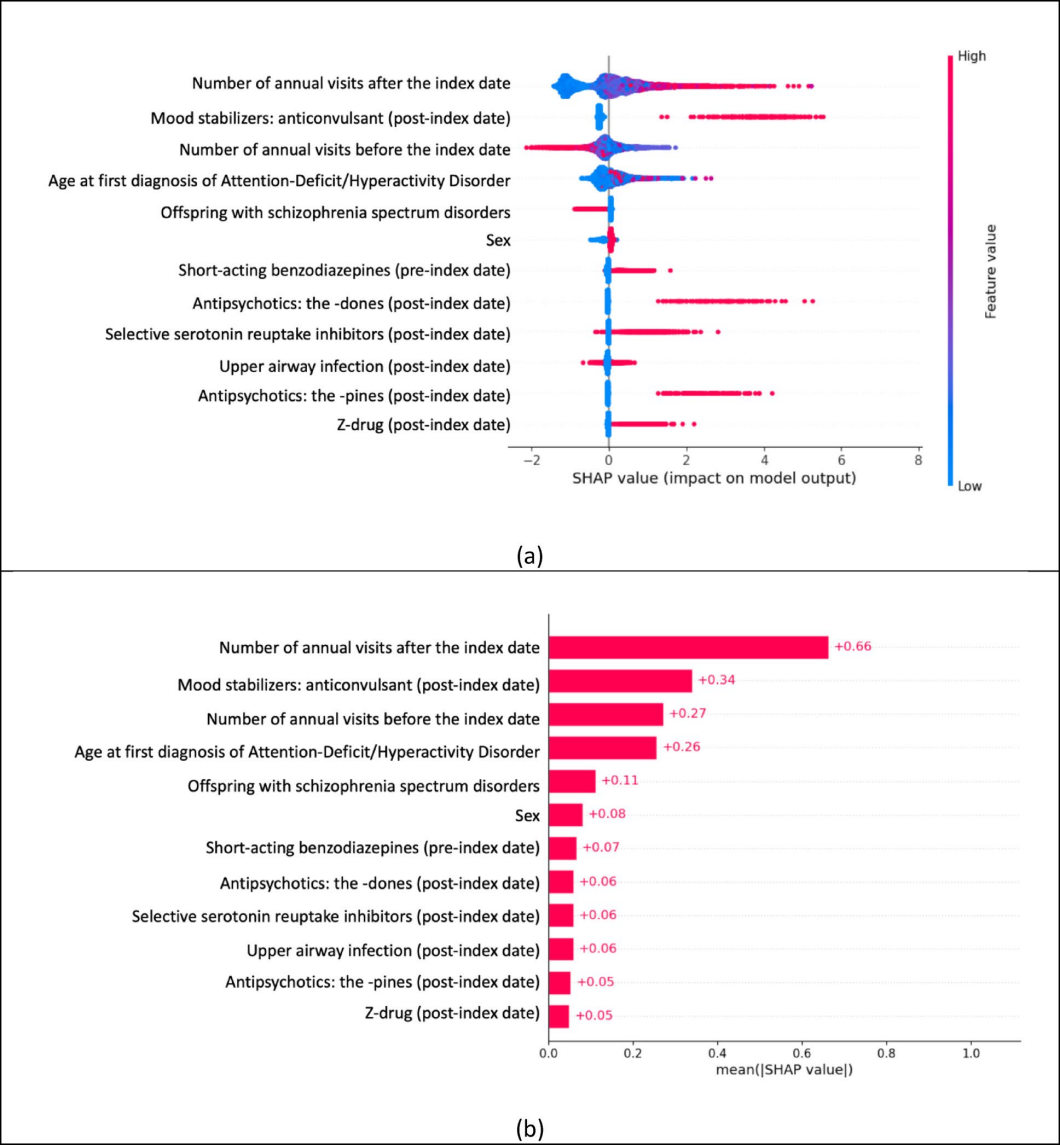
Shapley Additive Explanations (SHAP) method for the top 12 features (**a**) mean value; (**b**) the impact of value on model output

Figure 2a highlights the 12 predictors (approximately the top 5% of 217 features examined, each with a SHAP value ≥ 0.05) that were most strongly associated with the risk of BD comorbidity in patients with ADHD, whereas Fig. 2b depicts the direction and magnitude of every predictor’s contribution. The complete set of features is provided in eFigure 1. The healthcare utilization pattern characterized by infrequent psychiatric hospital visits before ADHD diagnosis (SHAP = 0.27) and higher frequency thereafter (SHAP = 0.66) emerged as the most influential behavioral predictor. Older age at ADHD diagnosis (SHAP = 0.26) and male sex (SHAP = 0.08) were the principal demographic risk factors to predict BD comorbidity. Pharmacological factors that amplified risk included pre-ADHD-diagnosis use of short-acting benzodiazepines (SHAP = 0.07) and post-ADHD-diagnosis exposure to anticonvulsant mood stabilizers (SHAP = 0.34), second-generation antipsychotics with generic names ending in “-done” (SHAP = 0.06) or “-pine” (SHAP = 0.05), selective serotonin-reuptake inhibitors (SHAP = 0.06), and Z-drugs (SHAP = 0.05). Conversely, having offspring diagnosed with schizophrenia-spectrum disorders (SHAP = 0.11) and experiencing fewer new-onset upper respiratory infections after ADHD diagnosis (SHAP = 0.06) were both associated with a lower predicted risk of developing BD. In addition, the temporal-window sensitivity analysis showed that Ablation-PostOnly model outperformed Ablation-PreOnly model (ROC-AUC 0.89 vs. 0.70; eTable 5). The age-stratified sensitivity analysis revealed heterogeneity consistent with the BD onset window: within the 12–25-year stratum, higher age was associated with higher risk, whereas within the ≥ 30-year stratum, relatively younger age was associated with higher risk (eTable 5; eFigure 2).

## Discussion

Using nationwide real-world data from Taiwan, we developed an XGBoost model to predict the five-year emergence of BD in patients with ADHD. In the independent test set, the model achieved a ROC-AUC of 0.90, an overall accuracy of 98%, a specificity of 99%, and a sensitivity of 50%. SHAP indicated several features that increased predicted risk: an outpatient-care pattern marked by relatively fewer psychiatric visits before ADHD diagnosis and more visits afterward; older age at ADHD diagnosis; male sex; pre-diagnosis use of short-acting benzodiazepines; post-diagnosis exposure to anticonvulsants (mood stabilizers), second-generation antipsychotics ending in “-dones” or “-pines”, and selective serotonin-reuptake inhibitors (antidepressants). By contrast, having offspring diagnosed with schizophrenia spectrum disorders and having fewer new-onset upper respiratory infections after ADHD diagnosis were associated with lower predicted risk.

In our cohort, only about 2% of patients with ADHD had comorbid BD during follow-up, a figure markedly lower than those reported elsewhere. A study using a decade of electronic health records from Johns Hopkins Medicine found an 11% conversion rate over 10 years [30], whereas a population-based follow-up study in Taiwan reported a 5% rate over 11 years [2]. This discrepancy is likely driven, at least in part, by study-design differences: constraints in our claims database restricted observation to a maximum of five years, whereas the other investigations tracked participants for considerably longer periods. Furthermore, in the temporal-window sensitivity analysis, models restricted to post-ADHD diagnosis features (Ablation-PostOnly) outperformed those restricted to pre-ADHD diagnosis features (Ablation-PreOnly) on ROC-AUC (0.89 vs. 0.70), indicating that post-index information carries greater predictive signal for near-term BD risk.

Psychiatric outpatient visit frequency measured before and after the ADHD index date emerged as two salient predictors of subsequent BD. Visit frequency serves as a pragmatic proxy for mental-health service utilization. In our data, these temporal features conveyed complementary signal: pre-index frequency reflected baseline context, whereas post-index frequency robustly signaled emerging clinical complexity or instability and was associated with increased subsequent BD risk. Prior research shows that ADHD complicated by BD is linked to poorer psychosocial functioning and a heavier burden of co-occurring psychiatric conditions [31]. This complexity calls for more intensive clinical engagement [32], requiring clinicians to see patients more often to manage emerging comorbidities. Fluctuating or irritable mood—initially interpreted as part of ADHD—may in fact represent prodromal manifestations of BD, warranting closer monitoring and more frequent evaluations [7]. In our cohort, older age at ADHD diagnosis was associated with a higher likelihood of subsequent BD. The mean diagnostic age was 19 years, and incident BD clustered thereafter, consistent with the typical peak onset between ages 17 and 26 [28, 29]. Thus, the “older age” signal is best interpreted as a proximity marker to the BD risk window rather than an independent etiologic driver. Evidence on sex-specific risk is mixed: some cohorts report greater progression in males [33], whereas others observe higher risk in females [2, 34]. In our data, the predictive model assigned higher risk to male patients with ADHD. Several interacting mechanisms may underlie this pattern. First, females with ADHD more often present with internalizing comorbidities (e.g., depression, anxiety), whereas males more frequently exhibit externalizing comorbidities (e.g., substance use disorders) [35]. This divergence may influence diagnostic attribution, with women’s mood symptoms more often classified as unipolar conditions that do not meet BD criteria, while men’s psychopathology is judged more consistent with bipolar-spectrum presentations [36]. Second, males are typically diagnosed with ADHD earlier and are more likely to receive stimulant therapy [37, 38]; observational reports suggest stimulant exposure may be associated with earlier BD onset in susceptible individuals, although confounding by indication and reverse causation could not be excluded [38]. In sum, sex differences in comorbidity profiles and treatment trajectories—together with the historical under-research of ADHD in females and differential ascertainment—may partly account for the higher male risk estimates. Definitive clarification will require prospective studies with standardized phenotyping and detailed treatment data.

Prescription of anticonvulsant mood stabilizers, second-generation antipsychotics with generic names ending in “-done” or “-pine,” or SSRI-type antidepressants after an ADHD diagnosis was associated with a subsequent diagnosis of BD. Use of mood stabilizers or antipsychotics typically signals greater clinical complexity in a subset of patients with ADHD, often manifested by impulse-control difficulties, hyperactivity or agitation, and other high-risk behaviors. Prior trials report that off-label use of divalproex or risperidone can attenuate aggression in ADHD [39, 40], and clinicians may introduce these agents to manage affective lability. Moreover, because such behavioral features can resemble hypomanic or manic presentations, their emergence may indicate prodromal bipolar pathology rather than severe ADHD per se. A similar rationale applies to antidepressant exposure: SSRI-induced mood switching is a well-recognized phenomenon, and in a longitudinal cohort of 22,797 youths with ADHD, treatment with fluoxetine, sertraline, trazodone, bupropion, or venlafaxine doubled the odds of a later BD [41]. A pediatric psychopharmacology review likewise emphasized that, although antidepressant-induced mania is uncommon overall, the risk is appreciably higher in ADHD populations and those with mood dysregulation [42], underscoring the need for vigilant monitoring when prescribing SSRIs in this group.

Our model found that ADHD probands whose offspring develop schizophrenia spectrum disorders are less likely to have comorbid BD themselves. Although uncommon, this signal was directionally consistent across all analytic datasets. At face value the finding appears counterintuitive given extensive cross-disorder pleiotropy among ADHD, schizophrenia, and BD [43], but pleiotropy is heterogeneous: multivariate genomic analyses delineate components that differentially load on psychosis- versus mania-linked liability, yielding partly separable polygenic profiles despite overlap [44]. Single nucleotide polymorphism-level colocalization further indicates that only a minority of ADHD loci are jointly shared with schizophrenia or with BD, implying substantial disorder-specific architecture [43]. Together with the enrichment in ADHD of neurodevelopmental copy number variations implicated in schizophrenia (e.g., 22q11.2, NRXN1) [45], these observations support a genetic-stratification hypothesis: ADHD–psychotic and ADHD–bipolar lineages may reflect partially distinct allele constellations. In such families, transmission of stronger schizophrenia-linked liability to offspring may index a relatively lower burden of BD-specific alleles in the proband, shifting trajectories away from affective outcomes and thereby reducing the proband’s subsequent BD risk. This interpretation is hypothesis-generating and warrants replication with independent cohorts and family-genetic data. Besides, immune–inflammatory load offers a complementary, modifiable pathway. In a prospective Taiwanese cohort, adolescents with ADHD plus asthma (a proxy for airway inflammation) had a three-fold increase in BD incidence compared with ADHD-only peers, whereas those without respiratory comorbidity showed the lowest risk [12]. A Danish nationwide study further demonstrated a dose-response rise in BD with the number of severe infections, including upper-airway episodes [46]. Collectively, these findings imply that maintaining a low respiratory-infection burden may attenuate manic emergence in genetically susceptible ADHD populations, suggesting infection prevention and prompt treatment as pragmatic secondary-prevention targets.

A major strength of this study is the use of a nationwide health-insurance claims database, which captures near-complete routine clinical practice in Taiwan and provides a robust real-world foundation for model development. However, several limitations must be acknowledged. First, because we relied on health insurance claims, key covariates such as educational attainment, socioeconomic status, and other potential confounders were unavailable, and family-relationship data drawn from the same source were sometimes incomplete. Second, the database’s temporal span restricted us to a uniform five-year follow-up for individuals diagnosed with ADHD at age ≥ 12 years; extending this observation window in future studies may uncover additional BD cases, thereby enabling more extensive reassessments of our predictors. Third, our claims-based case definition likely captured predominantly overt (mania-dominated) presentations of BD; subtler forms—especially bipolar II disorders, which may not be diagnosed until many years after initial depressive symptoms [47]—were probably under-represented. Fourth, several medications prescribed after the ADHD diagnosis (anticonvulsant mood stabilizers and second-generation antipsychotics) were major predictors of subsequent comorbid BD. Although off-label use supported by evidence is permissible in Taiwan, we cannot exclude confounding by indication—prescribers may have initiated these agents in response to emerging prodromal bipolar symptoms. Future work could interrogate this pathway using mediation analysis or clinician-suspected BD proxies. Fifth, despite using an independent test set to reduce overfitting and enhance generalizability, the cohort consisted solely of Taiwanese residents, largely reflecting East-Asian demographics. Consequently, external validation across diverse ethnic and geographic populations is essential before the model’s broader applicability can be confirmed. Sixth, while discrimination was strong (ROC-AUC 0.90) and specificity high (99%), sensitivity was modest (50%), indicating good rule-out performance but limited ability to detect true cases at the chosen threshold. Finally, the dataset exhibited substantial class imbalance (training subset total sample = 10,866; BD cases = 185). Although we applied class-imbalance weighting (balance between positive and negative classes = 59) during training, residual imbalance likely contributed to the gap between specificity and sensitivity.

## Conclusions

In a nationwide real-world cohort, our model predicted five-year incident comorbid BD in ADHD and yielded actionable signals. For example, in routine care, high-risk alerts based on increased post-diagnosis mental-health service use and post-diagnosis medication footprints (anticonvulsant mood stabilizers; “-done”/“-pine” second-generation antipsychotics; selective serotonin-reuptake inhibitors; Z-drugs) can prioritize closer follow-up, targeted bipolar screening, safety planning, and medication review, while diagnostic decisions remain with clinicians. Together, these patterns trace a clinically recognizable trajectory in which escalating care engagement and symptom-directed prescribing precede diagnosis, supporting the model as a pragmatic risk-stratification aid. Moreover, exploratory protective signals (e.g., fewer new-onset respiratory infections; having an offspring with schizophrenia-spectrum disorders) are hypothesis-generating and warrant prospective validation in independent cohorts and diverse settings.

## Supplementary Information

Supplementary Material 1.

## Data Availability

The data that support the findings of this study are available from the Taiwan National Health Insurance Research Database (NHIRD) but restrictions apply to the availability of these data, which were used under license for the current study, and so are not publicly available. Data are available from the authors upon reasonable request and with permission of the Taiwan National Health Insurance Administration.

## Abbreviations

ADHD: Attention-Deficit/Hyperactivity Disorder
ROC-AUC: Area Under the Receiver-Operating-Characteristic Curve
PR-AUC: Area Under the Precision-Recall Curve
PPV: Positive Predictive Value
BD: Bipolar Disorder
ICD-9-CM: International Classification of Diseases, Ninth Revision, Clinical Modification
NHIRD: Taiwan National Health Insurance Research Database
SHAP: Shapley Additive Explanations
SSRI: Selective Serotonin-Reuptake Inhibitor
XGBoost: Extreme Gradient Boosting
