# Supplementary material for: Predicting five-year comorbid bipolar disorder after attention-deficit/hyperactivity disorder diagnosis: a population-based machine learning approach
Source: Child Adolesc Psychiatry Ment Health. 2025 Dec 1;20:2. doi: 10.1186/s13034-025-01002-3 (PMC12776958; doi:10.1186/s13034-025-01002-3)
Supplement: Supplementary file 1 — Supplementary Material 1. [file 13034_2025_1002_MOESM1_ESM.docx]

**Supplementary material**

**Predicting Five-Year Comorbid Bipolar Disorder After Attention-Deficit/Hyperactivity Disorder Diagnosis: A Population-Based Machine Learning Approach**

**Running Title**: Predicting BD comorbidity in ADHD

Yen-Shan Yang, MD^a^, Chih-Wei Hsu, MD^a*^, Liang-Jen Wang, MD^b^, Kuo-Chuan Hung, MD^c^, Yang-Chieh Brian Chen, MD^d^, Chih-Sung Liang, MD^e,f^, Mu-Hong Chen, MD^g,h*^

^a^ Department of Psychiatry, Kaohsiung Chang Gung Memorial Hospital and Chang Gung University College of Medicine, Kaohsiung, Taiwan

^b^ Department of Child and Adolescent Psychiatry, Kaohsiung Chang Gung Memorial Hospital, Chang Gung University College of Medicine, Kaohsiung, Taiwan

^c^ Department of Anesthesiology, Chi Mei Medical Center, Tainan, Taiwan

^d^ Department of Psychiatry and Behavioral Sciences, The University of Texas Health Science Center at Houston, Houston, TX, USA

^e^ Department of Psychiatry, Beitou branch, Tri-Service General Hospital, National Defense Medical University, Taipei, Taiwan

^f^ Department of Psychiatry, National Defense Medical University, Taipei, Taiwan

^g^ Department of Psychiatry, Taipei Veterans General Hospital, Taipei, Taiwan

^h^ Department of Psychiatry, College of Medicine, National Yang Ming Chiao Tung University, Taipei, Taiwan

^*^**Contributed equally as corresponding authors**

| **Content** | **Page** |
| --- | --- |
| **eTable 1.** Detailed codification for drug names and diagnostic codes for mental disorders or medical conditions | 1-3 |
| **eTable 2.** Full list of the 217 features and their definitions | 4-11 |
| **eTable 3.** Characteristics of the development and test dataset, stratified by comorbid bipolar disorder diagnosis | 12 |
| **eTable 4.** Performance metrics of the predictive model across training, validation, and test set | 13 |
| **eTable 5.** Performance metrics of the predictive model using different temporal ablations or age-stratified methods | 14 |
| **eFigure 1.** Shapley Additive Explanations (SHAP) method for all features | 15-18 |
| **eFigure 2.** Shapley Additive Explanations (SHAP) method by age stratum, using impact on model output | 19 |

**eTable 1.** Detailed codification for drug names and diagnostic codes for mental disorders or medical conditions

| Drug name | Detailed drugs and their Anatomical Therapeutic Chemical (ATC) codes |
| --- | --- |
| Concomitant psychotropic medications |  |
| Mood stabilizers |  |
| Lithium | Lithium (N05AN01) |
| Anticonvulsants | Carbamazepine (N03AF01), Valproic acid (N03AG01), Lamotrigine (N03AX09), Topiramate (N03AX11) |
| Antidepressants |  |
| Selective serotonin reuptake inhibitors | Fluoxetine (N06AB03), Citalopram (N06AB04), Paroxetine (N06AB05), Sertraline (N06AN06), Fluvoxamine (N06AB08), Escitalopram (N06AB10) |
| Serotonin norepinephrine reuptake inhibitors | Venlafaxine (N06AX16), Milnacipran (N06AX17), Duloxetine (N06AX21), Desvenlafaxine (N06AX23), Levomilnacipran (N06AX28) |
| Serotonin antagonist and reuptake inhibitors | Trazodone (N06AX05) |
| Noradrenergic and specific serotonergic antidepressants | Mirtazapine (N06AX11) |
| Monoamine oxidase inhibitors | Isocarboxazid (N06AF01), Phenelzine (N06AF03), Tranylcypromine (N06AF04), Moclobemide (N06AG02) |
| Tricyclic antidepressants | Mianserin (N06AX03) |
| Norepinephrine and dopamine reuptake inhibitors | Bupropion (N06AX12) |
| Norepinephrine reuptake inhibitors | Viloxazine (N06AX09) |
| Melatonin agonists | Agomelatine (N06AX22) |
| Others | Oxitriptan (N06AX01)  Vortioxetine (N06AX26) |
| Antipsychotics |  |
| First-generation | Chlorpromazine (N05AA01), Levomepromazine (N05AA02), Fluphenazine (N05AB02), Perphenazine (N05AB03), Prochlorperazine (N05AB04), Trifluoperazine (N05AB06), Thioridazine (N05AC02), Pipotiazine (N05AC04), Haloperidol (N05AD01), Moperone (N05AD04), Droperidol (N05AD08), Flupentixol (N05AF01), Clopenthixol (N05AF02), Chlorprothixene (N05AF03), Tiotixene (N05AF04), Zuclopenthixol (N05AF05), Pimozide (N05AG02), Penfluridol (N05AG03), Loxapine (N05AH01), Clotiapine (N05AH06)  Zuclopenthixol (N05AF05) |
| The benzamides | Sulpiride (N05AL01), Amisulpride (N05AL05) |
| The -dones | Ziprasidone (N05AE04), Lurasidone (N05AE05), Risperidone (N05AX08), Paliperidone (N05AX13) |
| The -pines | Clozapine (N05AH02), Olanzapine (N05AH03), Quetiapine (N05AH04), Zotepine (N05AX11) |
| The -prazoles | Aripiprazole (N05AX12), Brexpiprazole (N05AX16) |
| Anxiolytics, sedatives, or hypnotics |  |
| Long-acting benzodiazepines | Clonazepam (N03AE01), Diazepam (N05BA01), Chlordiazepoxide (N05BA02), Medazepam (N05BA03), Bromazepam (N05BA08), Clobazam (N05BA09), Prazepam (N05BA11), Nordazepam (N05BA16), Cloxazolam (N05BA22), Nitrazepam (N05CD02), Flunitrazepam (N05CD03), Brotizolam (N05CD09) |
| Short-acting benzodiazepines | Oxazepam (N05BA04), Potassium clorazepate (N05BA05), Lorazepam (N05BA06), Alprazolam (N05BA12), Fludiazepam (N05BA17), Flurazepam (N05CD01),Estazolam (N05CD04), Triazolam (N05CD05), Lormetazepam (N05CD06), Temazepam (N05CD07), Midazolam (N05CD08), Nimetazepam (N05CD15) |
| Z-drugs | Zopiclone (N05CF01), Zolpidem (N05CF02), Zaleplon (N05CF03), Eszopiclone (N05CF04) |
| Others | Buspirone (N05BE01) |
| Mental disorders / Medical diseases | **The International Classification of Diseases 9^th^ (ICD-9)** |
| Mental disorders |  |
| Attention-deficit/hyperactivity disorder | 314 |
| Disruptive, impulse-control, and conduct disorders | 312.32, 312.33, 312.34, 312.8, 312.9, 313.81 |
| Schizophrenia spectrum disorders | 295, 297.0, 297.1, 297.2, 298.3, 298.4, 298.8, 298.9 |
| Bipolar disorders | 296.0, 296.1, 296.4–296.7, 296.80, 296.81, 296.89 |
| Depressive disorders | 296.2, 296.3, 296.99, 300.4, 311, 625.4 |
| Anxiety disorders | 300.00, 300.2, 309.21, 313.23 |
| Obsessive-compulsive and related disorders | 300.3, 312.39, 698.4 |
| Somatic symptom and related disorders | 300.11, 300.19, 300.7, 300.8, 316 |
| Elimination disorders | 307.6, 307.7, 787.60, 788.30, 788.39 |
| Sleep disorders | 307.4, 327, 347, 780.5 |
| Sexual dysfunctions | 302.7 |
| Substance-related and addictive disorders | 291.4, 291.81, 292.0, 292.2, 303, 304, 305, 312.31 |
| Neurocognitive disorders | 290.1, 290.2, 290.3, 294.1, 294.2, 331.0, 331.1, 331.2, 331.8, 331.9 |
| Personality disorders | 301.0, 301.20, 301.22, 301.4, 301.50, 301.6, 301.7, 301.81, 301.82, 301.83, 301.89, 301.9 |
| Trauma- and stressor-related disorders | 308.3, 309.0, 309.24, 309.28, 309.3, 309.4, 309.81, 309.89, 309.9, 313.89 |
| Dissociative disorders | 300.12, 300.13, 300.14, 300.15, 300.6 |
| Feeding and eating disorders | 307.1, 307.5 |
| Gender dysphoria | 302.6, 302.85 |
| Paraphilic disorders | 302.2, 302.3, 302.4, 302.81, 302.82, 302.83, 302.84, 302.89, 302.9 |
| Medical diseases |  |
| Myocardial infarct | 410, 412 |
| Congestive heart failure | 398.91, 402.01, 402.11, 402.91, 404.11, 404.13, 404.91, 404.93, 425.4, 425.5, 425.6, 425.7, 425.8, 425.9, 428 |
| Peripheral vascular diseases | 047.1, 093.0, 437.3, 440, 441, 443, 557.1, 557.9 |
| Cerebrovascular diseases | 362.34, 430, 431, 432, 433, 434, 435, 436, 437, 438 |
| Upper respiratory infection | 460 |
| Pneumonia | 480, 481, 482, 483, 484, 485, 486, 507 |
| Asthma | 493 |
| Chronic obstructive pulmonary disease | 490, 491, 492, 496 |
| Irritable bowel syndrome | 564.1 |
| Peptic ulcer diseases | 531, 532, 533, 534 |
| Liver diseases | 070.22, 070.23, 070.32, 070.33, 070.44, 070.54, 070.6, 070.9, 456.0, 456.1, 456.2, 570, 571, 572.2, 572.3, 572.4, 572.5, 572.6, 572.7, 572.8, 573.3, 573.4, 573.8 |
| Renal diseases | 403.01, 403.11, 403.91, 404.02, 404.03, 404.12, 404.13, 404.92, 404.93, 582, 583.0, 583.1, 583.2, 583.3, 583.4, 583.5, 583.6, 583.7, 585, 586, 588.0 |
| Connective tissue diseases | 710 |
| Cancer | 140, 141, 142, 143, 144, 145, 146, 147, 148, 149, 150, 151, 152, 153, 154, 155, 156, 157, 158, 159, 160, 161, 162, 163, 164, 165, 166, 167, 168, 169, 170, 171, 172, 174, 175, 176, 177, 178, 179, 180, 181, 182, 183, 184, 185, 186, 187, 188, 189, 190, 191, 192, 193, 194, 195, 195.0, 195.1, 195.2, 195.3, 195.4, 195.5, 195.6, 195.7, 195.8, 196, 197, 198, 199, 200, 201, 202, 203, 204, 205, 206, 207, 208, 238.6 |
| Acquired immune deficiency syndrome | 042, 043, 044 |
| Hypertension | 401, 402, 403, 404, 405 |
| Hyperlipidemia | 272 |
| Diabetes mellitus | 250.0, 250.1, 250.2, 250.3, 250.4, 250.5, 250.6, 250.7, 250.8, 250.9, |
| Atopic dermatitis | 691 |

**eTable 2.** Full list of the 217 features and their definitions

| Feature | Definition |
| --- | --- |
| Age | Age at index date |
| Sex | Biological sex of the patient (male or female) |
| Number of hospitalizations(Pre–index date) | Number of hospitalizations within 1 year prior to the index date |
| Number of hospitalizations(Post–index date) | Number of hospitalizations within 1 year after the index date |
| Number of outpatient visits(Pre–index date) | Number of outpatient visits within 1 year prior to the index date |
| Number of outpatient visits(Post–index date) | Number of outpatient visits within 1 year after the index date |
| Somatic symptom and related disorders (Pre–index date) | Presence of Somatic symptom and related disorders within 1 year before the index date until the index date (Pre–index date). |
| Somatic symptom and related disorders (Post–index date) | Presence of Somatic symptom and related disorders within 1 year after the index date until the end of observation (Post–index date). |
| Elimination disorders (Pre–index date) | Presence of Elimination disorders within 1 year before the index date until the index date (Pre–index date). |
| Elimination disorders (Post–index date) | Presence of Elimination disorders within 1 year after the index date until the end of observation (Post–index date). |
| Sleep–wake disorders (Pre–index date) | Presence of Sleep–wake disorders within 1 year before the index date until the index date (Pre–index date). |
| Sleep–wake disorders (Post–index date) | Presence of Sleep–wake disorders within 1 year after the index date until the end of observation (Post–index date). |
| Sexual dysfunctions (Pre–index date) | Presence of Sexual dysfunctions within 1 year before the index date until the index date (Pre–index date). |
| Sexual dysfunctions (Post–index date) | Presence of Sexual dysfunctions within 1 year after the index date until the end of observation (Post–index date). |
| Substance-related and addictive disorders (Pre–index date) | Presence of Substance-related and addictive disorders within 1 year before the index date until the index date (Pre–index date). |
| Substance-related and addictive disorders (Post–index date) | Presence of Substance-related and addictive disorders within 1 year after the index date until the end of observation (Post–index date). |
| Personality disorders (Pre–index date) | Presence of Personality disorders within 1 year before the index date until the index date (Pre–index date). |
| Personality disorders (Post–index date) | Presence of Personality disorders within 1 year after the index date until the end of observation (Post–index date). |
| Trauma- and stressor-related disorders (Pre–index date) | Presence of Trauma- and stressor-related disorders within 1 year before the index date until the index date (Pre–index date). |
| Trauma- and stressor-related disorders (Post–index date) | Presence of Trauma- and stressor-related disorders within 1 year after the index date until the end of observation (Post–index date). |
| Dissociative disorders (Pre–index date) | Presence of Dissociative disorders within 1 year before the index date until the index date (Pre–index date). |
| Dissociative disorders (Post–index date) | Presence of Dissociative disorders within 1 year after the index date until the end of observation (Post–index date). |
| Feeding and eating disorders (Pre–index date) | Presence of Feeding and eating disorders within 1 year before the index date until the index date (Pre–index date). |
| Feeding and eating disorders (Post–index date) | Presence of Feeding and eating disorders within 1 year after the index date until the end of observation (Post–index date). |
| Gender dysphoria (Pre–index date) | Presence of Gender dysphoria within 1 year before the index date until the index date (Pre–index date). |
| Gender dysphoria (Post–index date) | Presence of Gender dysphoria within 1 year after the index date until the end of observation (Post–index date). |
| Paraphilic disorders (Pre–index date) | Presence of Paraphilic disorders within 1 year before the index date until the index date (Pre–index date). |
| Paraphilic disorders (Post–index date) | Presence of Paraphilic disorders within 1 year after the index date until the end of observation (Post–index date). |
| Myocardial infarct (Pre–index date) | Presence of Myocardial infarct within 1 year before the index date until the index date (Pre–index date). |
| Myocardial infarct (Post–index date) | Presence of Myocardial infarct within 1 year after the index date until the end of observation (Post–index date). |
| Congestive heart failure (Pre–index date) | Presence of Congestive heart failure within 1 year before the index date until the index date (Pre–index date). |
| Congestive heart failure (Post–index date) | Presence of Congestive heart failure within 1 year after the index date until the end of observation (Post–index date). |
| Peripheral vascular diseases (Pre–index date) | Presence of Peripheral vascular diseases within 1 year before the index date until the index date (Pre–index date). |
| Peripheral vascular diseases (Post–index date) | Presence of Peripheral vascular diseases within 1 year after the index date until the end of observation (Post–index date). |
| Cerebrovascular diseases (Pre–index date) | Presence of Cerebrovascular diseases within 1 year before the index date until the index date (Pre–index date). |
| Cerebrovascular diseases (Post–index date) | Presence of Cerebrovascular diseases within 1 year after the index date until the end of observation (Post–index date). |
| Upper respiratory infection (Pre–index date) | Presence of Upper respiratory infection within 1 year before the index date until the index date (Pre–index date). |
| Upper respiratory infection (Post–index date) | Presence of Upper respiratory infection within 1 year after the index date until the end of observation (Post–index date). |
| Pneumonia (Pre–index date) | Presence of Pneumonia within 1 year before the index date until the index date (Pre–index date). |
| Pneumonia (Post–index date) | Presence of Pneumonia within 1 year after the index date until the end of observation (Post–index date). |
| Asthma (Pre–index date) | Presence of Asthma within 1 year before the index date until the index date (Pre–index date). |
| Asthma (Post–index date) | Presence of Asthma within 1 year after the index date until the end of observation (Post–index date). |
| Chronic obstructive pulmonary disease (Pre–index date) | Presence of Chronic obstructive pulmonary disease within 1 year before the index date until the index date (Pre–index date). |
| Chronic obstructive pulmonary disease (Post–index date) | Presence of Chronic obstructive pulmonary disease within 1 year after the index date until the end of observation (Post–index date). |
| Schizophrenia spectrum and other psychotic disorders (Pre–index date) | Presence of Schizophrenia spectrum and other psychotic disorders within 1 year before the index date until the index date (Pre–index date). |
| Schizophrenia spectrum and other psychotic disorders (Post–index date) | Presence of Schizophrenia spectrum and other psychotic disorders within 1 year after the index date until the end of observation (Post–index date). |
| Irritable bowel syndrome (Pre–index date) | Presence of Irritable bowel syndrome within 1 year before the index date until the index date (Pre–index date). |
| Irritable bowel syndrome (Post–index date) | Presence of Irritable bowel syndrome within 1 year after the index date until the end of observation (Post–index date). |
| Peptic ulcer diseases (Pre–index date) | Presence of Peptic ulcer diseases within 1 year before the index date until the index date (Pre–index date). |
| Peptic ulcer diseases (Post–index date) | Presence of Peptic ulcer diseases within 1 year after the index date until the end of observation (Post–index date). |
| Liver diseases (Pre–index date) | Presence of Liver diseases within 1 year before the index date until the index date (Pre–index date). |
| Liver diseases (Post–index date) | Presence of Liver diseases within 1 year after the index date until the end of observation (Post–index date). |
| Renal diseases (Pre–index date) | Presence of Renal diseases within 1 year before the index date until the index date (Pre–index date). |
| Renal diseases (Post–index date) | Presence of Renal diseases within 1 year after the index date until the end of observation (Post–index date). |
| Connective tissue disorders (Pre–index date) | Presence of Connective tissue disorders within 1 year before the index date until the index date (Pre–index date). |
| Connective tissue disorders (Post–index date) | Presence of Connective tissue disorders within 1 year after the index date until the end of observation (Post–index date). |
| Rheumatoid arthritis and other inflammatory polyarthropathies (Pre–index date) | Presence of Rheumatoid arthritis and other inflammatory polyarthropathies within 1 year before the index date until the index date (Pre–index date). |
| Rheumatoid arthritis and other inflammatory polyarthropathies (Post–index date) | Presence of Rheumatoid arthritis and other inflammatory polyarthropathies within 1 year after the index date until the end of observation (Post–index date). |
| Cancer (Pre–index date) | Presence of Cancer within 1 year before the index date until the index date (Pre–index date). |
| Cancer (Post–index date) | Presence of Cancer within 1 year after the index date until the end of observation (Post–index date). |
| Acquired immune deficiency syndrome (Pre–index date) | Presence of Acquired immune deficiency syndrome within 1 year before the index date until the index date (Pre–index date). |
| Acquired immune deficiency syndrome (Post–index date) | Presence of Acquired immune deficiency syndrome within 1 year after the index date until the end of observation (Post–index date). |
| Hypertension (Pre–index date) | Presence of Hypertension within 1 year before the index date until the index date (Pre–index date). |
| Hypertension (Post–index date) | Presence of Hypertension within 1 year after the index date until the end of observation (Post–index date). |
| Hyperlipidemia (Pre–index date) | Presence of Hyperlipidemia within 1 year before the index date until the index date (Pre–index date). |
| Hyperlipidemia (Post–index date) | Presence of Hyperlipidemia within 1 year after the index date until the end of observation (Post–index date). |
| Diabetes mellitus (Pre–index date) | Presence of Diabetes mellitus within 1 year before the index date until the index date (Pre–index date). |
| Diabetes mellitus (Post–index date) | Presence of Diabetes mellitus within 1 year after the index date until the end of observation (Post–index date). |
| Atopic dermatitis (Pre–index date) | Presence of Atopic dermatitis within 1 year before the index date until the index date (Pre–index date). |
| Atopic dermatitis (Post–index date) | Presence of Atopic dermatitis within 1 year after the index date until the end of observation (Post–index date). |
| Depressive disorders (Pre–index date) | Presence of Depressive disorders within 1 year before the index date until the index date (Pre–index date). |
| Depressive disorders (Post–index date) | Presence of Depressive disorders within 1 year after the index date until the end of observation (Post–index date). |
| Dementia (Pre–index date) | Presence of Dementia within 1 year before the index date until the index date (Pre–index date). |
| Dementia (Post–index date) | Presence of Dementia within 1 year after the index date until the end of observation (Post–index date). |
| Disruptive, impulse-control, and conduct disorders (Pre–index date) | Presence of Disruptive, impulse-control, and conduct disorders within 1 year before the index date until the index date (Pre–index date). |
| Disruptive, impulse-control, and conduct disorders (Post–index date) | Presence of Disruptive, impulse-control, and conduct disorders within 1 year after the index date until the end of observation (Post–index date). |
| Anxiety disorders (Pre–index date) | Presence of Anxiety disorders within 1 year before the index date until the index date (Pre–index date). |
| Anxiety disorders (Post–index date) | Presence of Anxiety disorders within 1 year after the index date until the end of observation (Post–index date). |
| Obsessive-compulsive and related disorders (Pre–index date) | Presence of Obsessive-compulsive and related disorders within 1 year before the index date until the index date (Pre–index date). |
| Obsessive-compulsive and related disorders (Post–index date) | Presence of Obsessive-compulsive and related disorders within 1 year after the index date until the end of observation (Post–index date). |
| Prescription of Other antidepressants (Pre–index date) | At least one prescription of Other antidepressants within 1 year before the index date until the index date (Pre–index date). |
| Prescription of Other antidepressants (Post–index date) | At least one prescription of Other antidepressants within 1 year after the index date until the end of observation (Post–index date). |
| Prescription of Selective serotonin reuptake inhibitors (Pre–index date) | At least one prescription of Selective serotonin reuptake inhibitors within 1 year before the index date until the index date (Pre–index date). |
| Prescription of Selective serotonin reuptake inhibitors (Post–index date) | At least one prescription of Selective serotonin reuptake inhibitors within 1 year after the index date until the end of observation (Post–index date). |
| Prescription of Serotonin norepinephrine reuptake inhibitors (Pre–index date) | At least one prescription of Serotonin norepinephrine reuptake inhibitors within 1 year before the index date until the index date (Pre–index date). |
| Prescription of Serotonin norepinephrine reuptake inhibitors (Post–index date) | At least one prescription of Serotonin–norepinephrine reuptake inhibitors within 1 year after the index date until the end of observation (Post–index date). |
| Prescription of Serotonin antagonist and reuptake inhibitors (Pre–index date) | At least one prescription of Serotonin antagonist and reuptake inhibitors within 1 year before the index date until the index date (Pre–index date). |
| Prescription of Serotonin antagonist and reuptake inhibitors (Post–index date) | At least one prescription of Serotonin antagonist and reuptake inhibitors within 1 year after the index date until the end of observation (Post–index date). |
| Prescription of Noradrenergic and specific serotonergic antidepressants (Pre–index date) | At least one prescription of Noradrenergic and specific serotonergic antidepressants within 1 year before the index date until the index date (Pre–index date). |
| Prescription of Noradrenergic and specific serotonergic antidepressants (Post–index date) | At least one prescription of Noradrenergic and specific serotonergic antidepressants within 1 year after the index date until the end of observation (Post–index date). |
| Prescription of Monoamine oxidase inhibitors (Pre–index date) | At least one prescription of Monoamine oxidase inhibitors within 1 year before the index date until the index date (Pre–index date). |
| Prescription of Monoamine oxidase inhibitors (Post–index date) | At least one prescription of Monoamine oxidase inhibitors within 1 year after the index date until the end of observation (Post–index date). |
| Prescription of Tricyclic antidepressants (Pre–index date) | At least one prescription of Tricyclic antidepressants within 1 year before the index date until the index date (Pre–index date). |
| Prescription of Tricyclic antidepressants (Post–index date) | At least one prescription of Tricyclic antidepressants within 1 year after the index date until the end of observation (Post–index date). |
| Prescription of Norepinephrine and dopamine reuptake inhibitors (Pre–index date) | At least one prescription of Norepinephrine and dopamine reuptake inhibitors within 1 year before the index date until the index date (Pre–index date). |
| Prescription of Norepinephrine and dopamine reuptake inhibitors (Post–index date) | At least one prescription of Norepinephrine and dopamine reuptake inhibitors within 1 year after the index date until the end of observation (Post–index date). |
| Prescription of Norepinephrine reuptake inhibitors (Pre–index date) | At least one prescription of Norepinephrine reuptake inhibitors within 1 year before the index date until the index date (Pre–index date). |
| Prescription of Norepinephrine reuptake inhibitors (Post–index date) | At least one prescription of Norepinephrine reuptake inhibitors within 1 year after the index date until the end of observation (Post–index date). |
| Prescription of Melatonin agonists (Pre–index date) | At least one prescription of Melatonin agonists within 1 year before the index date until the index date (Pre–index date). |
| Prescription of Melatonin agonists (Post–index date) | At least one prescription of Melatonin agonists within 1 year after the index date until the end of observation (Post–index date). |
| Prescription of First-generation antipsychotics (Pre–index date) | At least one prescription of First-generation antipsychotics within 1 year before the index date until the index date (Pre–index date). |
| Prescription of First-generation antipsychotics (Post–index date) | At least one prescription of First-generation antipsychotics within 1 year after the index date until the end of observation (Post–index date). |
| Prescription of the Benzamides antipsychotics (Pre–index date) | At least one prescription of the Benzamides antipsychotics within 1 year before the index date until the index date (Pre–index date). |
| Prescription of the Benzamides antipsychotics (Post–index date) | At least one prescription of the Benzamides antipsychotics within 1 year after the index date until the end of observation (Post–index date). |
| Prescription of Second-generation antipsychotics (–dones) (Pre–index date) | At least one prescription of Second-generation antipsychotics (–dones) within 1 year before the index date until the index date (Pre–index date). |
| Prescription of Second-generation antipsychotics (–dones) (Post–index date) | At least one prescription of Second-generation antipsychotics (–dones) within 1 year after the index date until the end of observation (Post–index date). |
| Prescription of Second-generation antipsychotics (–pines) (Pre–index date) | At least one prescription of Second-generation antipsychotics (–pines) within 1 year before the index date until the index date (Pre–index date). |
| Prescription of Second-generation antipsychotics (–pines) (Post–index date) | At least one prescription of Second-generation antipsychotics (–pines) within 1 year after the index date until the end of observation (Post–index date). |
| Prescription of Second-generation antipsychotics (–prazoles) (Pre–index date) | At least one prescription of Second-generation antipsychotics (–prazoles) within 1 year before the index date until the index date (Pre–index date). |
| Prescription of Second-generation antipsychotics (–prazoles) (Post–index date) | At least one prescription of Second-generation antipsychotics (–prazoles) within 1 year after the index date until the end of observation (Post–index date). |
| Prescription of Lithium (Pre–index date) | At least one prescription of Lithium within 1 year before the index date until the index date (Pre–index date). |
| Prescription of Lithium (Post–index date) | At least one prescription of Lithium within 1 year after the index date until the end of observation (Post–index date). |
| Prescription of Anticonvulsants (Pre–index date) | At least one prescription of Anticonvulsants within 1 year before the index date until the index date (Pre–index date). |
| Prescription of Anticonvulsants (Post–index date) | At least one prescription of Anticonvulsants within 1 year after the index date until the end of observation (Post–index date). |
| Prescription of Long-acting benzodiazepines (Pre–index date) | At least one prescription of Long-acting benzodiazepines within the specified observation window. |
| Prescription of Long-acting benzodiazepines (Post–index date) | At least one prescription of Long-acting benzodiazepines within 1 year after the index date until the end of observation (Post–index date). |
| Prescription of Short-acting benzodiazepines (Pre–index date) | At least one prescription of Short-acting benzodiazepines within the specified observation window. |
| Prescription of Short-acting benzodiazepines (Post–index date) | At least one prescription of Short-acting benzodiazepines within 1 year after the index date until the end of observation (Post–index date). |
| Prescription of Z-drugs (Pre–index date) | At least one prescription of Z-drugs within 1 year before the index date until the index date (Pre–index date). |
| Prescription of Z-drugs (Post–index date) | At least one prescription of Z-drugs within 1 year after the index date until the end of observation (Post–index date). |
| Prescription of Other benzodiazepines (Pre–index date) | At least one prescription of Other benzodiazepines within 1 year before the index date until the index date (Pre–index date). |
| Prescription of Other benzodiazepines (Post–index date) | At least one prescription of Other benzodiazepines within 1 year after the index date until the end of observation (Post–index date). |
| Grandchildren with Schizophrenia spectrum and other psychotic disorders | Presence of Schizophrenia spectrum and other psychotic disorders in grandchildren within the specified observation window. |
| Grandchildren with Disruptive, impulse-control, and conduct disorders | Presence of Disruptive, impulse-control, and conduct disorders in grandchildren within the specified observation window. |
| Grandchildren with Anxiety disorders | Presence of Anxiety disorders in grandchildren within the specified observation window. |
| Grandchildren with Obsessive-compulsive and related disorders | Presence of Obsessive-compulsive and related disorders in grandchildren within the specified observation window. |
| Grandchildren with Somatic symptom and related disorders | Presence of Somatic symptom and related disorders in grandchildren within the specified observation window. |
| Grandchildren with Elimination disorders | Presence of Elimination disorders in grandchildren within the specified observation window. |
| Grandchildren with Sleep–wake disorders | Presence of Sleep–wake disorders in grandchildren within the specified observation window. |
| Grandchildren with Sexual dysfunctions | Presence of Sexual dysfunctions in grandchildren within the specified observation window. |
| Grandchildren with Substance-related and addictive disorders | Presence of Substance-related and addictive disorders in grandchildren within the specified observation window. |
| Grandchildren with Personality disorders | Presence of Personality disorders in grandchildren within the specified observation window. |
| Grandchildren with Trauma- and stressor-related disorders | Presence of Trauma- and stressor-related disorders in grandchildren within the specified observation window. |
| Grandchildren with Dissociative disorders | Presence of Dissociative disorders in grandchildren within the specified observation window. |
| Grandchildren with Feeding and eating disorders | Presence of Feeding and eating disorders in grandchildren within the specified observation window. |
| Grandchildren with Gender dysphoria | Presence of Gender dysphoria in grandchildren within the specified observation window. |
| Grandchildren with Paraphilic disorders | Presence of Paraphilic disorders in grandchildren within the specified observation window. |
| Grandchildren with Attention-deficit/hyperactivity disorder | Presence of Attention-deficit/hyperactivity disorder in grandchildren within the specified observation window. |
| Grandchildren with Bipolar disorders | Presence of Bipolar disorders in grandchildren within the specified observation window. |
| Grandchildren with Depressive disorders | Presence of Depressive disorders in grandchildren within the specified observation window. |
| Grandchildren with Dementia | Presence of Dementia in grandchildren within the specified observation window. |
| Offspring with Schizophrenia spectrum and other psychotic disorders | Presence of Schizophrenia spectrum and other psychotic disorders in offspring within the specified observation window. |
| Offspring with Disruptive, impulse-control, and conduct disorders | Presence of Disruptive, impulse-control, and conduct disorders in offspring within the specified observation window. |
| Offspring with Anxiety disorders | Presence of Anxiety disorders in offspring within the specified observation window. |
| Offspring with Obsessive-compulsive and related disorders | Presence of Obsessive-compulsive and related disorders in offspring within the specified observation window. |
| Offspring with Somatic symptom and related disorders | Presence of Somatic symptom and related disorders in offspring within the specified observation window. |
| Offspring with Elimination disorders | Presence of Elimination disorders in offspring within the specified observation window. |
| Offspring with Sleep–wake disorders | Presence of Sleep–wake disorders in offspring within the specified observation window. |
| Offspring with Sexual dysfunctions | Presence of Sexual dysfunctions in offspring within the specified observation window. |
| Offspring with Substance-related and addictive disorders | Presence of Substance-related and addictive disorders in offspring within the specified observation window. |
| Offspring with Personality disorders | Presence of Personality disorders in offspring within the specified observation window. |
| Offspring with Trauma- and stressor-related disorders | Presence of Trauma- and stressor-related disorders in offspring within the specified observation window. |
| Offspring with Dissociative disorders | Presence of Dissociative disorders in offspring within the specified observation window. |
| Offspring with Feeding and eating disorders | Presence of Feeding and eating disorders in offspring within the specified observation window. |
| Offspring with Gender dysphoria | Presence of Gender dysphoria in offspring within the specified observation window. |
| Offspring with Paraphilic disorders | Presence of Paraphilic disorders in offspring within the specified observation window. |
| Offspring with Attention-deficit/hyperactivity disorder | Presence of Attention-deficit/hyperactivity disorder in offspring within the specified observation window. |
| Offspring with Bipolar disorders | Presence of Bipolar disorders in offspring within the specified observation window. |
| Offspring with Depressive disorders | Presence of Depressive disorders in offspring within the specified observation window. |
| Offspring with Dementia | Presence of Dementia in offspring within the specified observation window. |
| Grandparents with Schizophrenia spectrum and other psychotic disorders | Presence of Schizophrenia spectrum and other psychotic disorders in grandparents within the specified observation window. |
| Grandparents with Disruptive, impulse-control, and conduct disorders | Presence of Disruptive, impulse-control, and conduct disorders in grandparents within the specified observation window. |
| Grandparents with Anxiety disorders | Presence of Anxiety disorders in grandparents within the specified observation window. |
| Grandparents with Obsessive-compulsive and related disorders | Presence of Obsessive-compulsive and related disorders in grandparents within the specified observation window. |
| Grandparents with Somatic symptom and related disorders | Presence of Somatic symptom and related disorders in grandparents within the specified observation window. |
| Grandparents with Elimination disorders | Presence of Elimination disorders in grandparents within the specified observation window. |
| Grandparents with Sleep–wake disorders | Presence of Sleep–wake disorders in grandparents within the specified observation window. |
| Grandparents with Sexual dysfunctions | Presence of Sexual dysfunctions in grandparents within the specified observation window. |
| Grandparents with Substance-related and addictive disorders | Presence of Substance-related and addictive disorders in grandparents within the specified observation window. |
| Grandparents with Personality disorders | Presence of Personality disorders in grandparents within the specified observation window. |
| Grandparents with Trauma- and stressor-related disorders | Presence of Trauma- and stressor-related disorders in grandparents within the specified observation window. |
| Grandparents with Dissociative disorders | Presence of Dissociative disorders in grandparents within the specified observation window. |
| Grandparents with Feeding and eating disorders | Presence of Feeding and eating disorders in grandparents within the specified observation window. |
| Grandparents with Gender dysphoria | Presence of Gender dysphoria in grandparents within the specified observation window. |
| Grandparents with Paraphilic disorders | Presence of Paraphilic disorders in grandparents within the specified observation window. |
| Grandparents with Attention-deficit/hyperactivity disorder | Presence of Attention-deficit/hyperactivity disorder in grandparents within the specified observation window. |
| Grandparents with Bipolar disorders | Presence of Bipolar disorders in grandparents within the specified observation window. |
| Grandparents with Depressive disorders | Presence of Depressive disorders in grandparents within the specified observation window. |
| Grandparents with Dementia | Presence of Dementia in grandparents within the specified observation window. |
| Parents with Schizophrenia spectrum and other psychotic disorders | Presence of Schizophrenia spectrum and other psychotic disorders in parents within the specified observation window. |
| Parents with Disruptive, impulse-control, and conduct disorders | Presence of Disruptive, impulse-control, and conduct disorders in parents within the specified observation window. |
| Parents with Anxiety disorders | Presence of Anxiety disorders in parents within the specified observation window. |
| Parents with Obsessive-compulsive and related disorders | Presence of Obsessive-compulsive and related disorders in parents within the specified observation window. |
| Parents with Somatic symptom and related disorders | Presence of Somatic symptom and related disorders in parents within the specified observation window. |
| Parents with Elimination disorders | Presence of Elimination disorders in parents within the specified observation window. |
| Parents with Sleep–wake disorders | Presence of Sleep–wake disorders in parents within the specified observation window. |
| Parents with Sexual dysfunctions | Presence of Sexual dysfunctions in parents within the specified observation window. |
| Parents with Substance-related and addictive disorders | Presence of Substance-related and addictive disorders in parents within the specified observation window. |
| Parents with Personality disorders | Presence of Personality disorders in parents within the specified observation window. |
| Parents with Trauma- and stressor-related disorders | Presence of Trauma- and stressor-related disorders in parents within the specified observation window. |
| Parents with Dissociative disorders | Presence of Dissociative disorders in parents within the specified observation window. |
| Parents with Feeding and eating disorders | Presence of Feeding and eating disorders in parents within the specified observation window. |
| Parents with Gender dysphoria | Presence of Gender dysphoria in parents within the specified observation window. |
| Parents with Paraphilic disorders | Presence of Paraphilic disorders in parents within the specified observation window. |
| Parents with Attention-deficit/hyperactivity disorder | Presence of Attention-deficit/hyperactivity disorder in parents within the specified observation window. |
| Parents with Bipolar disorders | Presence of Bipolar disorders in parents within the specified observation window. |
| Parents with Depressive disorders | Presence of Depressive disorders in parents within the specified observation window. |
| Parents with Dementia | Presence of Dementia in parents within the specified observation window. |
| Siblings with Schizophrenia spectrum and other psychotic disorders | Presence of Schizophrenia spectrum and other psychotic disorders in siblings within the specified observation window. |
| Siblings with Disruptive, impulse-control, and conduct disorders | Presence of Disruptive, impulse-control, and conduct disorders in siblings within the specified observation window. |
| Siblings with Anxiety disorders | Presence of Anxiety disorders in siblings within the specified observation window. |
| Siblings with Obsessive-compulsive and related disorders | Presence of Obsessive-compulsive and related disorders in siblings within the specified observation window. |
| Siblings with Somatic symptom and related disorders | Presence of Somatic symptom and related disorders in siblings within the specified observation window. |
| Siblings with Elimination disorders | Presence of Elimination disorders in siblings within the specified observation window. |
| Siblings with Sleep–wake disorders | Presence of Sleep–wake disorders in siblings within the specified observation window. |
| Siblings with Sexual dysfunctions | Presence of Sexual dysfunctions in siblings within the specified observation window. |
| Siblings with Substance-related and addictive disorders | Presence of Substance-related and addictive disorders in siblings within the specified observation window. |
| Siblings with Personality disorders | Presence of Personality disorders in siblings within the specified observation window. |
| Siblings with Trauma- and stressor-related disorders | Presence of Trauma- and stressor-related disorders in siblings within the specified observation window. |
| Siblings with Dissociative disorders | Presence of Dissociative disorders in siblings within the specified observation window. |
| Siblings with Feeding and eating disorders | Presence of Feeding and eating disorders in siblings within the specified observation window. |
| Siblings with Gender dysphoria | Presence of Gender dysphoria in siblings within the specified observation window. |
| Siblings with Paraphilic disorders | Presence of Paraphilic disorders in siblings within the specified observation window. |
| Siblings with Attention-deficit/hyperactivity disorder | Presence of Attention-deficit/hyperactivity disorder in siblings within the specified observation window. |
| Siblings with Bipolar disorders | Presence of Bipolar disorders in siblings within the specified observation window. |
| Siblings with Depressive disorders | Presence of Depressive disorders in siblings within the specified observation window. |
| Siblings with Dementia | Presence of Dementia in siblings within the specified observation window. |

**eTable 3.** Characteristics of the development and test dataset, stratified by comorbid bipolar disorder diagnosis

| Characteristics | Development | | | Test | | |
| --- | --- | --- | --- | --- | --- | --- |
|  | Training (n = 10,866) | | Validation (n = 2717) | | Test (n = 1510) | |
|  | No comorbid  (n = 10,681) | Comorbid BD  (n = 185) | No comorbid  (n = 2666) | Comorbid BD  (n = 51) | No comorbid  (n = 1480) | Comorbid BD  (n = 30) |
| Basic information |  |  |  |  |  |  |
| Age at first diagnosis of ADHD | 18.6 ± 9.9 | 22.8 ± 12.1 | 18.8 ± 10.0 | 19.8 ± 6.7 | 18.1 ± 9.3 | 21.4 ± 9.2 |
| Sex, female | 3209 (30.0) | 67 (36.2) | 809 (30.3) | 16 (31.4) | 445 (30.1) | 12 (40.0) |
| Psychiatric outpatient visit |  |  |  |  |  |  |
| Number of annual visits before the index date | 13.5 ± 10.2 | 16.8 ± 12.1 | 13.3 ± 10.5 | 20.1 ± 15.4 | 13.3 ± 10.0 | 14.4 ± 8.3 |
| Number of annual visits before the end date | 11.1 ± 10.4 | 28.6 ± 18.8 | 11.4 ± 10.6 | 27.7 ±1 8.5 | 10.9 ± 10.2 | 28.4 ± 24.1 |
| Medical comorbidities (Pre–index date) |  |  |  |  |  |  |
| Upper respiratory infection | 1344 (12.6) | 20 (10.8) | 310 (11.6) | 10 (19.6) | 182 (12.3) | 2 (6.7) |
| Asthma | 283 (2.6) | 2 (1.1) | 73 (2.7) | 1 (2.0) | 44 (3.0) | 1 (3.3) |
| Psychiatric comorbidities (Pre–index date) |  |  |  |  |  |  |
| Depressive disorders | 464 (4.3) | 48 (25.9) | 111 (4.2) | 14 (27.5) | 49 (3.3) | 11 (36.7) |
| Anxiety disorders | 300 (2.8) | 22 (11.9) | 80 (3.0) | 6 (11.8) | 34 (2.3) | 3 (10.0) |
| Medical comorbidities (Post–index date) |  |  |  |  |  |  |
| Upper respiratory infection | 1950 (18.3) | 29 (15.7) | 486 (18.2) | 12 (23.5) | 267 (18.0) | 3 (10.0) |
| Asthma | 412 (3.9) | 6 (3.2) | 99 (3.7) | 2 (3.9) | 61 (4.1) | 1 (3.3) |
| Psychiatric comorbidities (Post–index date) |  |  |  |  |  |  |
| Depressive disorders | 678 (6.3) | 66 (35.7) | 180 (6.8) | 25 (49.0) | 87 (5.9) | 14 (46.7) |
| Anxiety disorders | 586 (5.5) | 36 (19.5) | 138 (5.2) | 9 (17.6) | 69 (4.7) | 7 (23.3) |
| Psychotropic drugs (Pre–index date) |  |  |  |  |  |  |
| Mood stabilizers |  |  |  |  |  |  |
| Anticonvulsants | 233 (2.2) | 26 (14.1) | 43 (1.6) | 8 (15.7) | 27 (1.8) | 3 (10.0) |
| Lithium | 16 (0.1) | 7 (3.8) | 6 (0.2) | 0 (0.0) | 1 (0.1) | 3 (10.0) |
| Antipsychotics |  |  |  |  |  |  |
| First-generation | 494 (4.6) | 18 (9.7) | 118 (4.4) | 7 (13.7) | 51 (3.4) | 7 (23.3) |
| The benzamides | 381 (3.6) | 23 (12.4) | 104 (3.9) | 8 (15.7) | 46 (3.1) | 2 (6.7) |
| The -dones | 165 (1.5) | 9 (4.9) | 33 (1.2) | 2 (3.9) | 26 (1.8) | 3 (10.0) |
| The -pines | 60 (0.6) | 12 (6.5) | 10 (0.4) | 3 (5.9) | 9 (0.6) | 1 (3.3) |
| Antidepressants |  |  |  |  |  |  |
| Selective serotonin reuptake inhibitors | 1134 (10.6) | 71 (38.4) | 276 (10.4) | 24 (47.1) | 146 (9.9) | 13 (43.3) |
| Serotonin–norepinephrine reuptake inhibitors | 135 (1.3) | 18 (9.7) | 35 (1.3) | 2 (3.9) | 24 (1.6) | 1 (3.3) |
| Anxiolytics, sedatives, or hypnotics |  |  |  |  |  |  |
| Long-acting benzodiazepines | 1014 (9.5) | 47 (25.4) | 256 (9.6) | 20 (39.2) | 116 (7.8) | 6 (20.0) |
| Short-acting benzodiazepines | 1031 (9.7) | 67 (36.2) | 264 (9.9) | 20 (39.2) | 136 (9.2) | 12 (40.0) |
| Z-drugs | 469 (4.4) | 44 (23.8) | 120 (4.5) | 14 (27.5) | 63 (4.3) | 7 (23.3) |
| Psychotropic drugs (Post–index date) |  |  |  |  |  |  |
| Mood stabilizers |  |  |  |  |  |  |
| Anticonvulsants | 186 (1.7) | 93 (50.3) | 40 (1.5) | 32 (62.7) | 23 (1.6) | 11 (36.7) |
| Lithium | 9 (0.1) | 16 (8.6) | 1 (0.0) | 3 (5.9) | 0 (0.0) | 1 (3.3) |
| Antipsychotics |  |  |  |  |  |  |
| First-generation | 467 (4.4) | 20 (10.8) | 122 (4.6) | 7 (13.7) | 50 (3.4) | 6 (20.0) |
| The benzamides | 174 (1.6) | 30 (16.2) | 48 (1.8) | 12 (23.5) | 23 (1.6) | 4 (13.3) |
| The -dones | 87 (0.8) | 30 (16.2) | 27 (1.0) | 7 (13.7) | 17 (1.1) | 4 (13.3) |
| The -pines | 71 (0.7) | 43 (23.2) | 14 (0.5) | 8 (15.7) | 9 (0.6) | 3 (10.0) |
| Antidepressants |  |  |  |  |  |  |
| Selective serotonin reuptake inhibitors | 478 (4.5) | 78 (42.2) | 127 (4.8) | 31 (60.8) | 78 (5.3) | 15 (50) |
| Serotonin–norepinephrine reuptake inhibitors | 80 (0.7) | 19 (10.3) | 28 (1.1) | 6 (11.8) | 6 (0.4) | 2 (6.7) |
| Anxiolytics, sedatives, or hypnotics |  |  |  |  |  |  |
| Long-acting benzodiazepines | 752 (7.0) | 71 (38.4) | 194 (7.3) | 25 (49.0) | 100 (6.8) | 12 (40.0) |
| Short-acting benzodiazepines | 929 (8.7) | 91 (49.2) | 222 (8.3) | 26 (51.0) | 116 (7.8) | 14 (46.7) |
| Z-drugs | 345 (3.2) | 61 (33.0) | 93 (3.5) | 15 (29.4) | 45 (3.0) | 6 (20.0) |
| Psychiatric family history |  |  |  |  |  |  |
| Attention-deficit/hyperactivity disorder |  |  |  |  |  |  |
| Parents | 212 (2.0) | 2 (1.1) | 55 (2.1) | 0 (0.0) | 26 (1.8) | 0 (0.0) |
| Siblings | 1183 (11.1) | 11 (5.9) | 276 (10.4) | 2 (3.9) | 179 (12.1) | 4 (13.3) |
| Offspring | 1003 (9.4) | 11 (5.9) | 250 (9.4) | 0 (0.0) | 123 (8.3) | 1 (3.3) |
| Schizophrenia spectrum disorders |  |  |  |  |  |  |
| Parents | 148 (1.4) | 3(1.6) | 36 (1.4) | 3 (5.9) | 33 (2.2) | 1 (3.3) |
| Siblings | 97 (0.9) | 0 (0.0) | 22 (0.8) | 1 (2.0) | 23 (1.6) | 1 (3.3) |
| Offspring | 27 (0.3) | 0 (0.0) | 8 (0.3) | 0 (0.0) | 2 (0.1) | 0 (0.0) |
| Bipolar disorders |  |  |  |  |  |  |
| Parents | 140 (1.3) | 4 (2.2) | 38 (1.4) | 1 (2.0) | 26 (1.8) | 2 (6.7) |
| Siblings | 92 (0.9) | 2 (1.1) | 14 (0.5) | 3 (5.9) | 21 (1.4) | 1 (3.3) |
| Offspring | 20 (0.2) | 0 (0.0) | 3 (0.2) | 0 (0.0) | 3 (0.2) | 0 (0.0) |

^1^ Abbreviation: ADHD, attention-deficit/hyperactivity disorder; BD, bipolar disorder

^2^ Data was expressed as N (percentage) or mean ± standard deviation

**eTable 4.** Performance metrics of the predictive model across training, validation, and test set

| Dataset | ROC-AUC | PR-AUC | Accuracy | Specificity | Sensitivity | PPV |
| --- | --- | --- | --- | --- | --- | --- |
| Primary analysis | | | | | | |
| Training | 1.00 | 0.98 | 0.99 | 0.99 | 1.00 | 0.63 |
| Validation | 0.97 | 0.50 | 0.97 | 0.98 | 0.67 | 0.40 |
| Test | 0.90 | 0.59 | 0.98 | 0.99 | 0.50 | 0.43 |
| Sensitivity analysis | | | | | | |
| Training | 0.99 (0.98-0.99) | 0.83 (0.76-0.89) | 0.99 (0.99-0.99) | 0.99 (0.99-0.99) | 0.86 (0.81-0.91) | 0.67 (0.60-0.74) |
| Validation | 0.96 (0.95-0.97) | 0.49 (0.45-0.54) | 0.98 (0.98-0.98) | 0.99 (0.99-0.99) | 0.55 (0.49-0.60) | 0.46 (0.42-0.51) |
| Test | 0.92 (0.91-0.93) | 0.52 (0.50-0.54) | 0.98 (0.98-0.98) | 0.99 (0.99-0.99) | 0.49 (0.44-0.54) | 0.51 (0.48-0.54) |

Abbreviations: PPV, positive predictive value; PR-AUC, area under the precision–recall curve; ROC-AUC, area under the receiver operating characteristic curve

Sensitivity analysis data were expressed as mean and 95% confidence interval.

**eTable 5.** Performance metrics of the predictive model using different temporal ablations or age-stratified methods.

| Dataset | ROC-AUC | PR-AUC | Accuracy | Specificity | Sensitivity | PPV |
| --- | --- | --- | --- | --- | --- | --- |
| Sensitivity analysis 1: Ablation-PostOnly | | | | | | |
| Training | 0.98 | 0.60 | 0.94 | 0.94 | 0.93 | 0.22 |
| Validation | 0.97 | 0.59 | 0.94 | 0.94 | 0.90 | 0.23 |
| Test | 0.89 | 0.51 | 0.94 | 0.94 | 0.77 | 0.20 |
| Sensitivity analysis 1: Ablation-PreOnly | | | | | | |
| Training | 0.90 | 0.24 | 0.87 | 0.87 | 0.75 | 0.09 |
| Validation | 0.79 | 0.09 | 0.85 | 0.86 | 0.61 | 0.07 |
| Test | 0.70 | 0.16 | 0.85 | 0.85 | 0.50 | 0.06 |
| Sensitivity analysis 2: Age-Stratum-12–25 | | | | | | |
| Training | 0.99 | 0.80 | 0.97 | 0.97 | 0.97 | 0.30 |
| Validation | 0.98 | 0.47 | 0.96 | 0.96 | 0.78 | 0.28 |
| Test | 0.92 | 0.47 | 0.95 | 0.96 | 0.69 | 0.27 |
| Sensitivity analysis 2: Age-Stratum-≥30 | | | | | | |
| Training | 1.00 | 0.99 | 0.98 | 0.98 | 1.00 | 0.56 |
| Validation | 0.95 | 0.24 | 0.97 | 0.98 | 0.38 | 0.27 |
| Test | 0.99 | 0.65 | 0.98 | 0.99 | 0.88 | 0.70 |

Abbreviations: PPV, positive predictive value; PR-AUC, area under the precision–recall curve; ROC-AUC, area under the receiver operating characteristic curve

**eFigure 1.** Shapley Additive Explanations (SHAP) method for all features

a) SHAP value (mean)


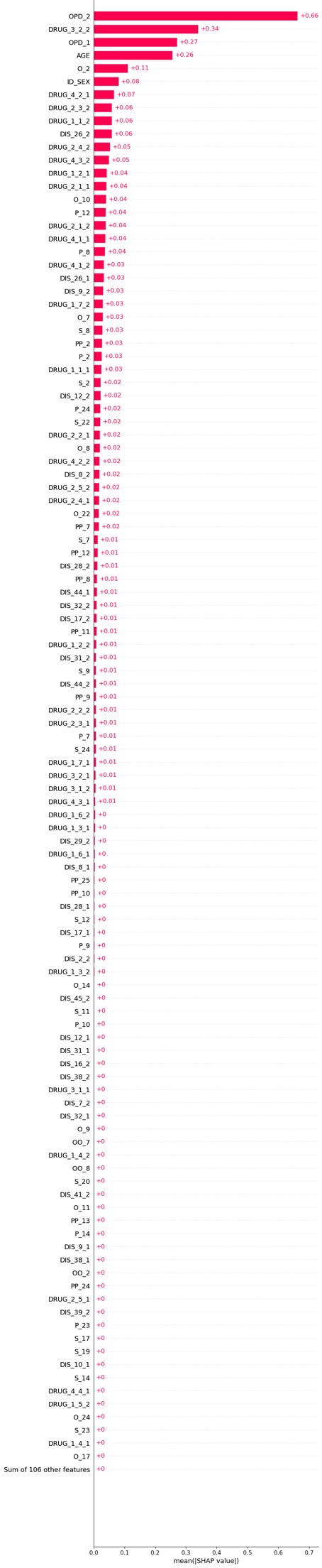


b) SHAP value (impact on model output)


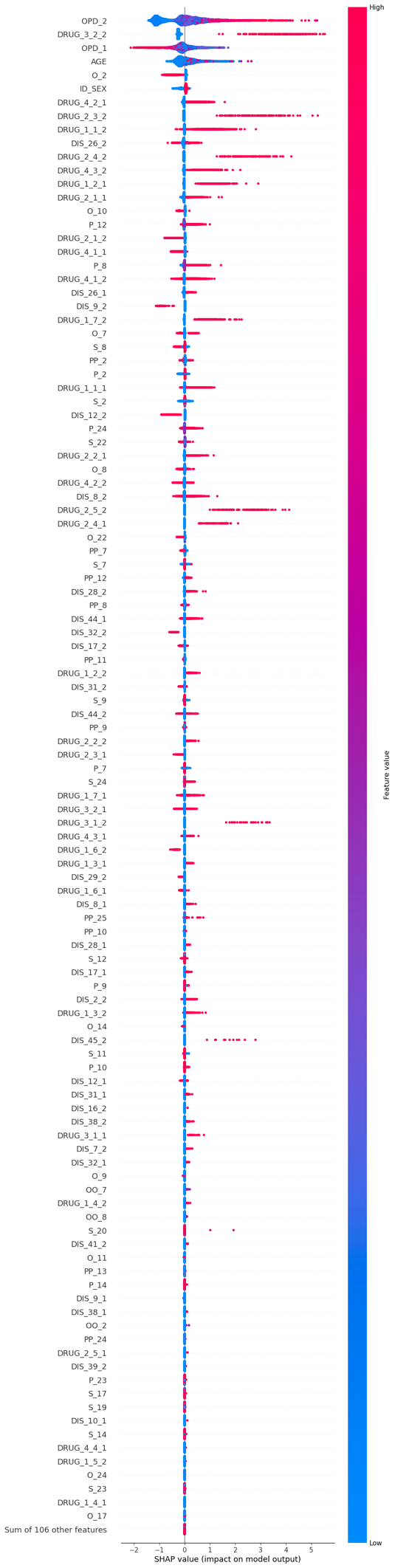


^1^ Abbreviation: OPD_2 = Number of annual visits after the index date, DRUG_3_2_2 = Mood stabilizers: anticonvulsants (post-index date), OPD_1 = Number of annual visits before the index date, AGE = Age at first diagnosis of Attention-Deficit/Hyperactivity Disorder, O_2 = Schizophrenia spectrum disorders (Children), ID_SEX = Sex, DRUG_4_2_1 = Short-acting benzodiazepines (pre-index date), DRUG_2_3_2 = Antipsychotics: the -dones (post-index date), DRUG_1_1_2 = Selective serotonin reuptake inhibitors (post-index date), DIS_26_2 = Upper airway infection (post-index date), DRUG_2_4_2 = Antipsychotics: the -pines (post-index date), DRUG_4_3_2 = Z-drugs (post-index date), DRUG_1_2_1 = Serotonin-norepinephrine reuptake inhibitors (pre-index date), DRUG_2_1_1 = Antipsychotics: 1st generation (pre-index date), O_10 = Somatic symptom and related disorders (Children), P_12 = Sleep-wake disorders (Parents), DRUG_2_1_2 = Antipsychotics: 1st generation (post-index date), DRUG_4_1_1 = Long-acting benzodiazepines (pre-index date), P_8 = Anxiety disorders (Parents), DRUG_4_1_2 = Long-acting benzodiazepines (post-index date), DIS_26_1 = Upper airway infection (pre-index date), DIS_9_2 = Obsessive-compulsive and related disorders (post-index date), DRUG_1_7_2 = Norepinephrine-dopamine reuptake inhibitors (post-index date), O_7 = Disruptive, impulse-control, and conduct disorders (Children), S_8 = Anxiety disorders (Siblings), PP_2 = Schizophrenia spectrum disorders (Grandparents), P_2 = Schizophrenia spectrum disorders (Parents), DRUG_1_1_1 = Selective serotonin reuptake inhibitors (pre-index date), S_2 = Schizophrenia spectrum disorders (Siblings), DIS_12_2 = Sleep-wake disorders (post-index date), P_24 = Depressive disorders (Parents), S_22 = Attention-Deficit/Hyperactivity Disorder (Siblings), DRUG_2_2_1 = Antipsychotics: The benzamides (pre-index date), O_8 = Anxiety disorders (Children), DRUG_4_2_2 = Short-acting benzodiazepines (post-index date), DIS_8_2 = Anxiety disorders (post-index date), DRUG_2_5_2 = Antipsychotics: the -prazoles (post-index date), DRUG_2_4_1 = Antipsychotics: the -pines (pre-index date), O_22 = Attention-Deficit/Hyperactivity Disorder (Children), PP_7 = Disruptive, impulse-control, and conduct disorders (Grandparents), S_7 = Disruptive, impulse-control, and conduct disorders (Siblings), PP_12 = Sleep-wake disorders (Grandparents), DIS_28_2 = Asthma (post-index date), PP_8 = Anxiety disorders (Grandparents), DIS_44_1 = Depressive disorders (pre-index date), DIS_32_2 = Liver diseases (post-index date), DIS_17_2 = Trauma- and stressor-related disorders (post-index date), PP_11 = Elimination disorders (Grandparents), DRUG_1_2_2 = Serotonin-norepinephrine reuptake inhibitors (post-index date), DIS_31_2 = Peptic ulcer diseases (post-index date), S_9 = Obsessive-compulsive and related disorders (Siblings), DIS_44_2 = Depressive disorders (post-index date), PP_9 = Obsessive-compulsive and related disorders (Grandparents), DRUG_2_2_2 = Antipsychotics: The benzamides (post-index date), DRUG_2_3_1 = Antipsychotics: the -dones (pre-index date), P_7 = Disruptive, impulse-control, and conduct disorders (Parents), S_24 = Depressive disorders (Siblings), DRUG_1_7_1 = Norepinephrine-dopamine reuptake inhibitors (pre-index date), DRUG_3_2_1 = Mood stabilizers: anticonvulsants (pre-index date), DRUG_3_1_2 = Mood stabilizers: lithium (post-index date), DRUG_4_3_1 = Z-drugs (pre-index date), DRUG_1_6_2 = Tricyclic antidepressants (post-index date), DRUG_1_3_1 = Serotonin antagonist and reuptake inhibitors (pre-index date), DIS_29_2 = COPD (post-index date), DRUG_1_6_1 = Tricyclic antidepressants (pre-index date), DIS_8_1 = Anxiety disorders (pre-index date), PP_25 = Dementia (Grandparents), PP_10 = Somatic symptom and related disorders (Grandparents), DIS_28_1 = Asthma (pre-index date), S_12 = Sleep-wake disorders (Siblings), DIS_17_1 = Trauma- and stressor-related disorders (pre-index date), P_9 = Obsessive-compulsive and related disorders (Parents), DIS_2_2 = Schizophrenia spectrum disorders (post-index date), DRUG_1_3_2 = Serotonin antagonist and reuptake inhibitors (post-index date), O_14 = Substance-related and addictive disorders (Children), DIS_45_2 = Dementia (post-index date), S_11 = Elimination disorders (Siblings), P_10 = Somatic symptom and related disorders (Parents), DIS_12_1 = Sleep-wake disorders (pre-index date), DIS_31_1 = Peptic ulcer diseases (pre-index date), DIS_16_2 = Personality disorders (post-index date), DIS_38_2 = Hypertension (post-index date), DRUG_3_1_1 = Mood stabilizers: lithium (pre-index date), DIS_7_2 = Disruptive, impulse-control, and conduct disorders (post-index date), DIS_32_1 = Liver diseases (pre-index date), O_9 = Obsessive-compulsive and related disorders (Children), OO_7 = Disruptive, impulse-control, and conduct disorders (Grandchildren), DRUG_1_4_2 = Noradrenergic and specific serotonergic antidepressants (post-index date), OO_8 = Anxiety disorders (Grandchildren), S_20 = Gender dysphoria (Siblings), DIS_41_2 = Atopic dermatitis (post-index date), O_11 = Elimination disorders (Children), PP_13 = Sexual dysfunctions (Grandparents), P_14 = Substance-related and addictive disorders (Parents), DIS_9_1 = Obsessive-compulsive and related disorders (pre-index date), DIS_38_1 = Hypertension (pre-index date), OO_2 = Schizophrenia spectrum disorders (Grandchildren), PP_24 = Depressive disorders (Grandparents), DRUG_2_5_1 = Antipsychotics: the -prazoles (pre-index date), DIS_39_2 = Hyperlipidemia (post-index date), P_23 = Bipolar disorders (Parents), S_17 = Trauma- and stressor-related disorders (Siblings), S_19 = Feeding and eating disorders (Siblings), DIS_10_1 = Somatic symptom and related disorders (pre-index date), S_14 = Substance-related and addictive disorders (Siblings), DRUG_4_4_1 = Other benzodiazepines (pre-index date), DRUG_1_5_2 = Monoamine oxidase inhibitors (post-index date), O_24 = Depressive disorders (Children), S_23 = Bipolar disorders (Siblings), DRUG_1_4_1 = Noradrenergic and specific serotonergic antidepressants (pre-index date), O_17 = Trauma- and stressor-related disorders (Children)

^2^ The bottom 106 features do not contribute SHAP value in the model

**eFigure 2.** Shapley Additive Explanations (SHAP) method by age stratum, using impact on model output

a) Age-Stratum-12–25


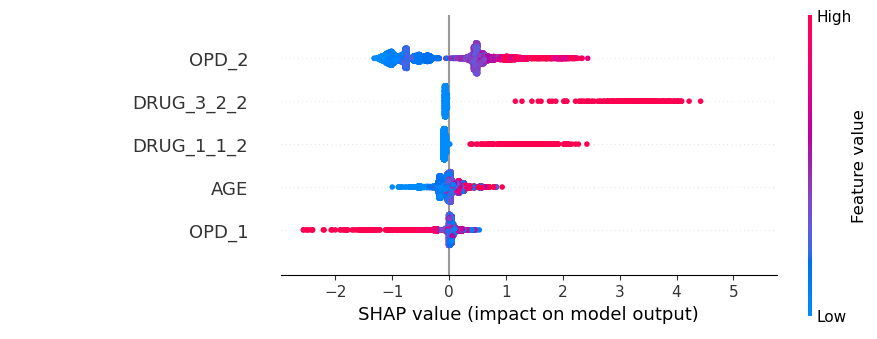


b) Age-Stratum-≥30


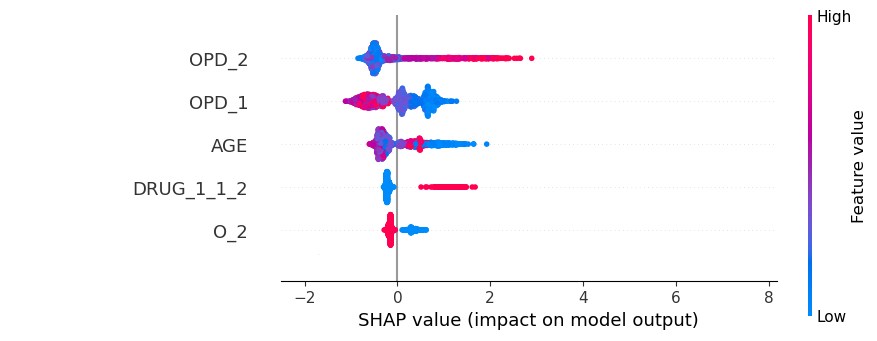


Abbreviation: OPD_2 = Number of annual visits after the index date, DRUG_3_2_2 = Mood stabilizers: anticonvulsants (post-index date), DRUG_1_1_2 = Selective serotonin reuptake inhibitors (post-index date), AGE = Age at first diagnosis of Attention-Deficit/Hyperactivity Disorder, OPD_1 = Number of annual visits before the index date, O_2 = Schizophrenia spectrum disorders (Children)
